# Adipose-derived stem cell exosomes: mechanisms and therapeutic potentials in wound healing

**DOI:** 10.1186/s40364-025-00801-2

**Published:** 2025-06-20

**Authors:** Heting Feng, Song Gong, Jiafeng Liu, Sis Aghayants, Yiling Liu, Min Wu, Yiping Wu, Jingyu Song

**Affiliations:** 1 Department of Plastic and Cosmetic Surgery, Tongji Hospital, Tongji Medical College, Huazhong University of Science and Technology, 1095 Jiefang Avenue, Wuhan, 430030 Hubei China; 2 Division of Endocrinology, Tongji Hospital, Huazhong University of Science and Technology, Jiefang Road 1095, Wuhan, 430030 Hubei Province People’s Republic of China; 3 Department of Plastic Surgery, Renmin Hospital of Wuhan University, Wuhan, 430060 Hubei Province China; 4 Medical Cosmetology Department, Hubei Aerospace Hospital, Xiaogan, 432001 Hubei China

**Keywords:** Wound healing, Adipose-derived stem cell, Exosome, Engineering, Angiogenesis, Therapeutic potential

## Abstract

Wound healing is a complex, multi-stage process that restores skin integrity through coordinated cellular and molecular interactions. Among the emerging therapeutic strategies, adipose-derived stem cell exosomes (ADSC-Exos) attract significant attention due to their potent regenerative capabilities. ADSC-Exos contribute to wound repair by modulating inflammatory responses, promoting cellular proliferation and migration, stimulating angiogenesis, and facilitating collagen remodeling. These exosomes carry a diverse array of bioactive molecules including cytokines, non-coding RNAs (ncRNAs), and proteins, that are delivered to target cells, thereby orchestrating the intricate processes involved in tissue regeneration. Recent advancements in exosome engineering, such as genetic modification, pharmacological preconditioning, hypoxic treatment, and incorporation with biomaterials, markedly improve the therapeutic efficacy of ADSC-Exos. This review summarizes the underlying mechanisms and therapeutic potential of ADSC-Exos in wound healing, offering new perspectives for developing exosome-based regenerative therapies. Nevertheless, challenges persist regarding the large-scale production, standardized isolation, and clinical translation of ADSC-Exos. Future research should aim to enhance exosome yield and purity, elucidate the mechanisms governing exosome biogenesis, and validate their clinical efficacy through well-designed trials.

## Introduction

**Fig. 1:**
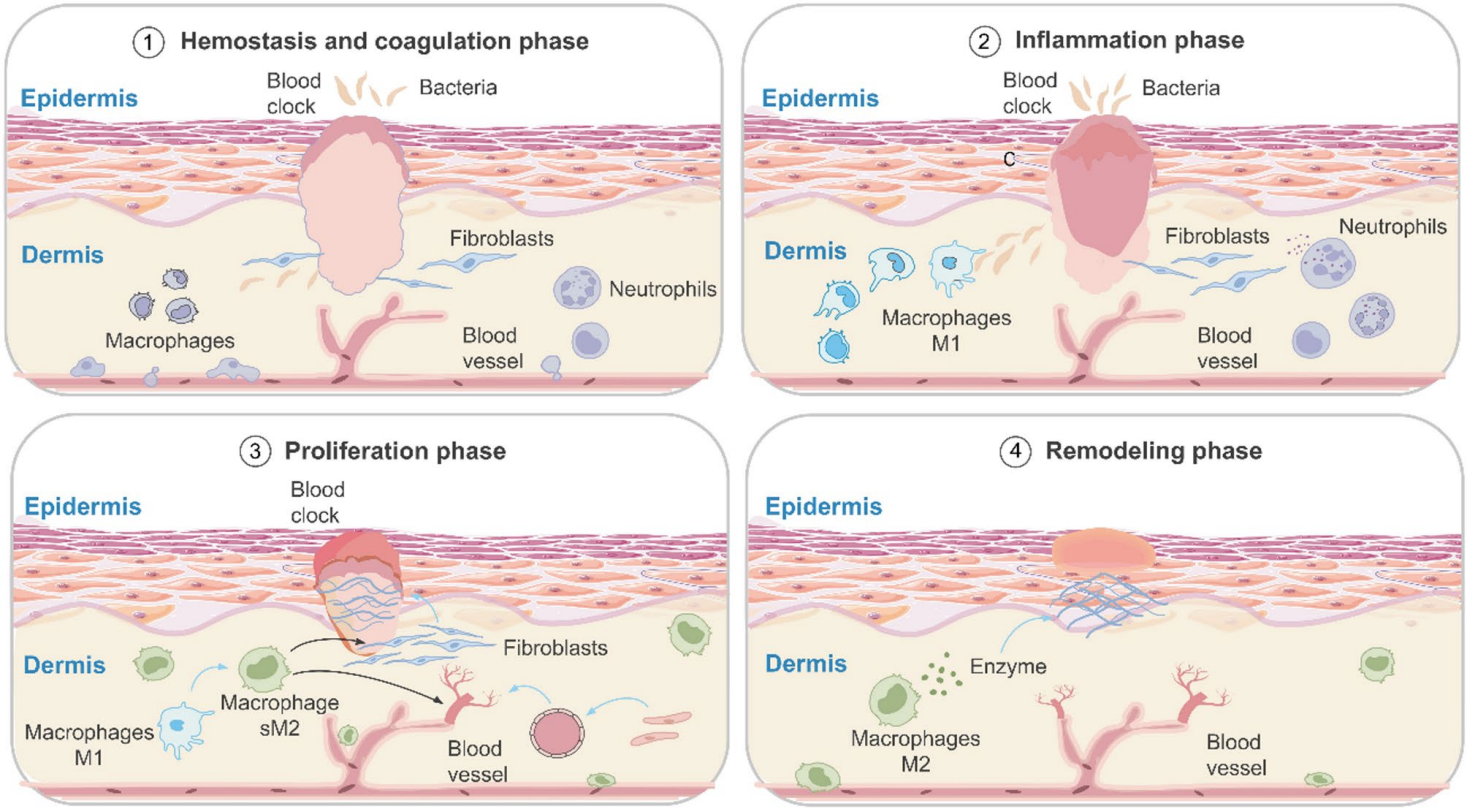
Four stages of normal wound healing. The wound healing process encompasses four sequential and overlapping phases: hemostasis and coagulation, inflammation, proliferation, and remodeling. Macrophages, fibroblasts, vascular endothelial cells, and other cells undergo proliferation, polarization, and various processes, releasing a wide range of cytokines that collectively constitute this event. Biogenesis, contents, biomarkers, and intercellular communication of exosomes. During exosome biogenesis, intraluminal vesicles (ILVs) and multivesicular bodies (MVBs) are formed by the invagination of the endosomal membrane. MVBs fuse with lysosomes to release ILVs, which are degraded in the lumen of the lysosomes, or with the plasma membrane to release exosomes. Exosomes are in the size range of 30 nm to 200 nm, with a specific subset of biomolecules, such as membrane proteins (CD63, CD9, Rab family), cytosolic proteins (HSP, KRAS, HMGB1), and major histocompatibility complexes (MHC-I, MHC-II). Exosomes also contain a diverse array of biologically active enzymes, lipids, mRNA, DNA, and non-coding RNAs (miRNAs, lncRNAs, circRNAs). For regulation of cellular functions, exosomes interact with the plasma membrane of recipient cells via the exosomal membrane or deliver biomolecules into the cells. Exosomes communicate with the recipient cell through direct fusion, receptor-ligand interactions, and endocytosis/phagocytosis

Skin wounds are disruptions in the structure and integrity of the skin tissue, resulting from various intrinsic pathological conditions or external mechanical factors [1]. Wound healing is initiated immediately after a skin injury, following a well-defined and sequential process aimed at restoring the skin’s barrier function. During the process, there are four overlapping and interdependent phases: hemostasis, inflammation, proliferation, and tissue remodeling [2]. The regulation of these phases is mediated by a complex interplay of cytokines, chemokines, and growth factors. Any disturbances in these molecular interactions can hinder the healing process and contribute to scar formation [3]. Failure of proper healing, or delayed healing of skin wounds, can lead to both local and systemic pathological consequences, causing significant pain and imposing a substantial economic burden on patients [4]. Various strategies have been explored to enhance wound healing, including diverse wound dressings [5], negative pressure suction [6], skin substitution therapy [7], flap grafting [8], and stem cell transplantation [9]. Despite the effectiveness of these interventions in optimizing wound care, challenges such as atrophic scarring, pigmentation abnormalities, and immune rejection persist [10] (Fig. 1).

Adipose tissue, the largest endocrine organ in the body, plays a pivotal role in regulating both metabolism and the immune system and is found throughout the body [11]. It is primarily composed of adipocytes and the stromal vascular fraction (SVF). The SVF consists of a heterogeneous population of cells, predominantly adipose-derived stem cells (ADSCs), along with various immune cells, fibroblasts, stromal cells, and vascular endothelial cells [12]. ADSCs are easily isolated from the SVF of liposuctioned subcutaneous adipose tissue, following collagenase digestion [13].

ADSCs are a population of multipotent stem cells with extensive differentiation potential, capable of differentiating into various cell types, including adipocytes, osteocytes, chondrocytes, myocytes, epithelial cells, and neuronal cells [14, 15]. ADSCs also possess the ability to secrete a range of paracrine factors, such as growth factors, cytokines, neurotrophic factors, chemokines, and vesicles [16]. Growth factors like vascular endothelial growth factor (VEGF) and fibroblast growth factor (FGF) promote angiogenesis and fibroblast proliferation, respectively, while platelet-derived growth factor (PDGF) enhances collagen synthesis and tissue remodeling. Cytokines, such as the anti-inflammatory interleukin-10 (IL-10) and transforming growth factor-β (TGF-β), help to suppress excessive inflammation and encourage extracellular matrix (ECM) deposition. In contrast, pro-inflammatory cytokines like tumor necrosis factor-α (TNF-α) and interferon-γ (IFN-γ) are downregulated during later stages to prevent chronic inflammation that could impair wound healing. Chemokines such as C-C motif chemokine ligand 2 (CCL2) and CXCL12 facilitate the recruitment of immune and stromal cells to the wound site, aiding in debris clearance and tissue regeneration. Together, these mediators coordinate cell recruitment, inflammation resolution, ECM remodeling, and re-epithelialization, all of which are essential for effective wound healing. Paracrine signaling represents the primary mechanism through which ADSCs contribute to tissue regeneration, stimulate angiogenesis, and modulate immune responses [17].

Extracellular vesicles (EVs) are cell-derived particles enclosed by lipid bilayers, which can be classified into two main types: exosomes and multivesicular vesicles, based on their origins [18]. Exosomes, ranging from 30 nm to 200 nm in diameter, are derived from the endosomes of eukaryotic cells [19]. There is considerable heterogeneity both between different exosome types and within individual types, with some overlap in features with other EVs [20]. Regardless of their origin, exosomes consistently contain a specific subset of cellular proteins, including tetraspanins (CD81, CD63, CD9), heat shock proteins (HSP70, HSP90), endosomal biogenesis-associated proteins (ALIX, TSG101), and major histocompatibility complexes (MHC I, MHC II) [21]. In addition to proteins, exosomes also carry a diverse array of biologically active and conserved substances, such as lipids and nucleic acids [22]. These substances play a crucial role in intercellular communication, being transferred to recipient cells and mediating various cellular functions [23]. The impact of exosomes is broad, influencing essential biological processes, such as cell proliferation, differentiation, metabolism, and apoptosis [24–26]. ADSC-Exos constitute a significant portion of the secretory products of ADSCs [27], carrying a range of biologically active molecules, and acting as key mediators of the therapeutic effects of ADSCs [28].

ADSC-Exos offer distinct advantages over exosomes derived from other sources, primarily due to their accessibility and inherent biological properties. Adipose tissue, which is widely distributed, and easily accessible through minimally invasive liposuction, contains a high number of ADSCs, with colony-forming units significantly surpassing those of bone marrow mesenchymal stem cells (BM-MSCs) [29]. This abundance of adipose tissue facilitates the large-scale production of ADSC-Exos, with fewer ethical constraints and simpler isolation procedures, compared to exosomes derived from cord MSCs or BMSCs [30]. ADSCs also exhibit robust proliferative capacity in vitro, ensuring efficient expansion and consistent exosome production, even in simplified culture systems supplemented with human platelet lysate. In contrast, other stem cell sources face scalability challenges due to donor variability or the onset of functional senescence during prolonged culture [31]. ADSC-Exos are characterized by low immunogenicity and an absence of tumorigenic risks, conferring a superior safety profile. Additionally, the autologous nature of adipose tissue minimizes the expression of MHC I/II on ADSC-Exos, significantly reducing the potential for immune rejection, compared to other sources of allogeneic exosomes [32]. Unlike exosomes derived from pluripotent or embryonic stem cells, ADSC-Exos eliminate concerns regarding teratoma formation. These factors collectively enhance productivity, batch consistency, and the therapeutic potential of ADSC-Exos in diverse clinical applications.

ADSC-Exos have demonstrated significant therapeutic potential in the treatment of various clinical conditions, particularly in tissue regeneration, including wound healing, bone tissue repair, and skin flap transplantation [33]. In the context of wound healing, ADSC-Exos have been shown to influence multiple stages of the healing process by delivering cytokines, ncRNAs, and other biologically active molecules [34]. Genetic modification of exosome contents [27], pre-treatment of ADSCs [35], and the integration of exosomes with biomaterials can enhance the reparative effects of ADSC-Exos [36]. Therefore, this review focuses on summarizing the therapeutic effects and underlying mechanisms of ADSC-Exos in wound healing. A deeper understanding of their biological properties in this context will facilitate the development of ADSC-Exos-based therapeutic strategies.

## Biogenesis of exosomes

Some initial insights have been gained into the process of exosome formation. The process begins with the inward budding of the endosomal membrane, followed by the formation of invaginations that are pinched off and released as intra-luminal vesicles (ILVs) within the endosome, resulting in the formation of multi-vesicular bodies (MVBs) [37]. MVBs can follow either the secretory or lysosomal pathway. In the secretory pathway, MVBs fuse with the plasma membrane, releasing their ILVs as exosomes, while the peripheral membrane of the MVB is incorporated into the cell membrane. In the lysosomal pathway, MVBs fuse with lysosomes, where ILVs are degraded within the lysosomal lumen.

During this process, exosomes selectively encapsulate specific proteins and lipids into the endosomal membrane. Monoubiquitination of the cytoplasmic domains of transmembrane proteins, whether internalized from the cell surface or translocated from the trans-Golgi network, serves as a crucial sorting signal that directs cargoes into ILVs. Additionally, the endosomal sorting complex required for transport (ESCRT) mechanism selectively recognizes and captures these ubiquitinated proteins [38]. However, accumulating evidence highlights the existence of multiple content-sorting pathways. For instance, tetraspanins can organize membrane microdomains that cluster specific cargoes, such as MHC II, in a manner independent of ubiquitination [22]. Lipid-mediated mechanisms, including ceramide-induced membrane curvature and sphingomyelinase activity, also promote ILV formation through ESCRT-independent routes. Moreover, Syndecan-1 and Syntenin-1 facilitate ubiquitination-independent sorting by recruiting the ALIX-ESCRT-III complex to direct cargoes into the ILVs [39]. Notably, MVBs can still form, even in the absence of core ESCRT subunits, highlighting the mechanistic flexibility and redundancy of exosome biogenesis pathways. This plasticity appears to be influenced by factors such as cell type, genomic stability, and extracellular stimuli. These findings indicate that exosome biogenesis is governed by a broad spectrum of mechanisms, many of which remain incompletely understood and warrant further investigation [40]. In summary, cargoes sorting into ILVs occurs through both ESCRT-dependent and ESCRT-independent mechanisms [41].

The selective incorporation of ncRNAs into ADSC-Exos is regulated by microenvironmental stress-induced interactions between RNA-binding proteins (RBPs) and specific structural motifs on the RNAs [42]. RBPs such as hnRNPA2B1 recognize GW/RGG exo-motifs on ncRNAs, directing their sorting into multivesicular bodies for subsequent exosomal packaging. Under hypoxic conditions, hypoxia-inducible factor-1α (HIF-1α) binds to hypoxia-response elements in the promoter region of the lncRNA NORAD, promoting its expression and exosomal export. Simultaneously, hypoxia-induced SUMOylation enhances hnRNPA2B1-mediated recruitment of miR-524-5p into exosomes [43]. Additionally, hypoxia stabilizes AUF1 through USP22-mediated deubiquitination, allowing AUF1 to bind AU-rich elements in lncRNA H19, facilitating its exosomal inclusion while promoting the degradation of pro-inflammatory mRNAs such as TNF-α [44]. Oxidative stress further modulates exosomal cargo selection via post-translational modifications. For example, reactive oxygen species (ROS)-induced acetylation of SYNCRIP enhances its affinity for conserved GAUC motifs in microRNAs (miRNAs) like let-7i-5p, promoting their incorporation into exosomes [45]. This regulatory network highlights a dynamic interplay in which stress signals influence RBP activity and RNA motif recognition, enabling the context-dependent packaging of anti-inflammatory and reparative ncRNAs. Moreover, complementary interactions between miRNAs and circular RNAs (circRNAs) contribute to their co-sorting into exosomes. Together, these coordinated mechanisms ensure that ADSC-Exos are selectively enriched with ncRNAs tailored to mitigate injury-related pathways, thereby optimizing their therapeutic potential in inflammatory microenvironments.

**Fig. 2:**
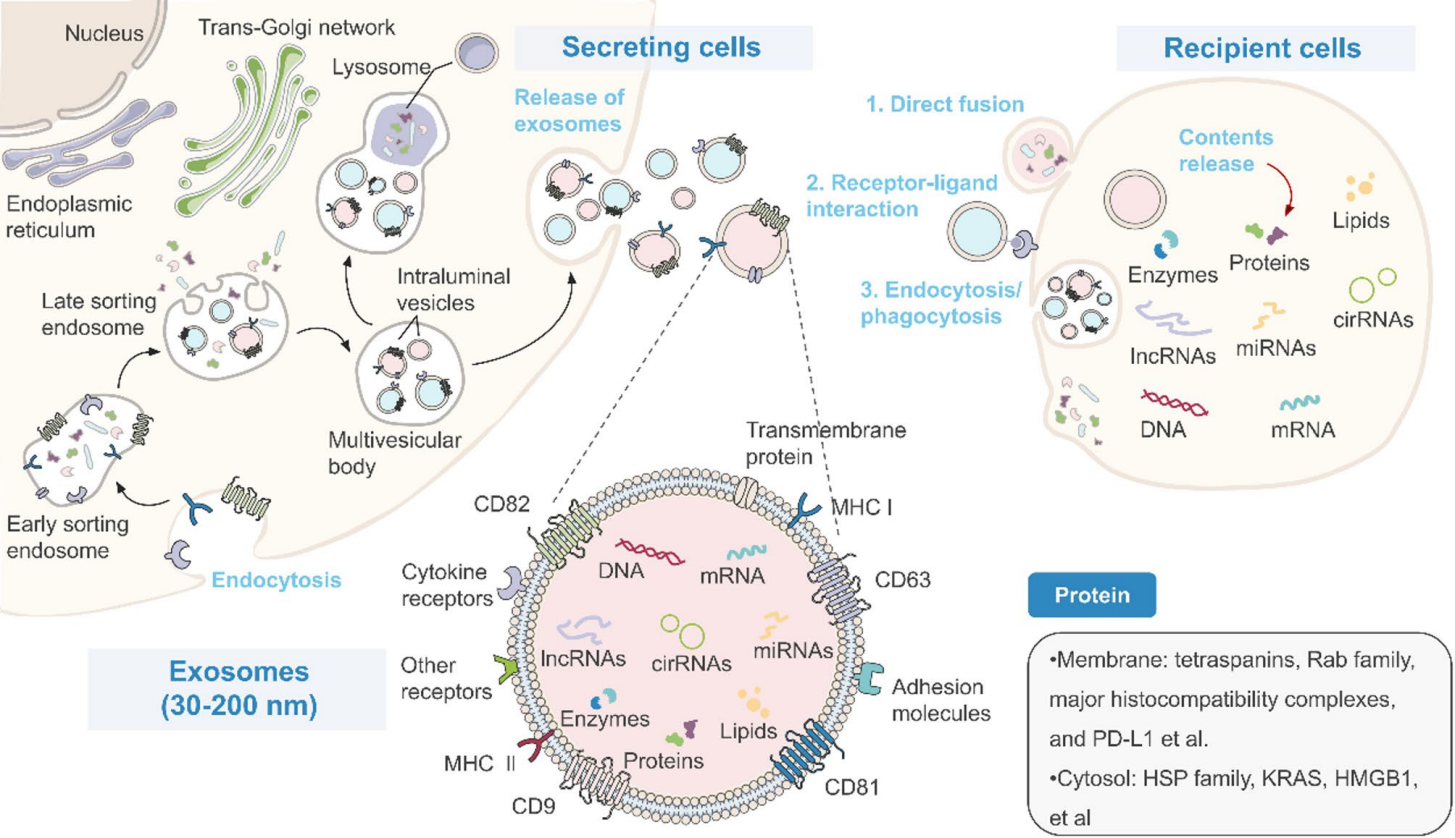
Biogenesis, contents, biomarkers, and intercellular communication of exosomes. During exosome biogenesis, intraluminal vesicles (ILVs) and multivesicular bodies (MVBs) are formed by the invagination of the endosomal membrane. MVBs fuse with lysosomes to release ILVs, which are degraded in the lumen of the lysosomes, or with the plasma membrane to release exosomes. Exosomes are in the size range of 30 nm to 200 nm, with a specific subset of biomolecules, such as membrane proteins (CD63, CD9, Rab family), cytosolic proteins (HSP, KRAS, HMGB1), and major histocompatibility complexes (MHC-I, MHC-II). Exosomes also contain a diverse array of biologically active enzymes, lipids, mRNA, DNA, and non-coding RNAs (miRNAs, lncRNAs, circRNAs). For regulation of cellular functions, exosomes interact with the plasma membrane of recipient cells via the exosomal membrane or deliver biomolecules into the cells. Exosomes communicate with the recipient cell through direct fusion, receptor-ligand interactions, and endocytosis/phagocytosis

After secretion, exosomes can adhere to neighboring cells and the ECM or disseminate to distant sites via bodily fluids such as blood. Previous studies have identified three primary mechanisms through which exosomes transmit signals to recipient cells: receptor-ligand interactions, direct membrane fusion, and endocytosis or phagocytosis [46]. Exosomes derived from various cell types such as blood cells, endothelial cells, immune cells, platelets, and smooth muscle cells play key roles in immune responses, tumor progression, and neurodegenerative diseases [47]. The release of exosomes and their interaction with target cells are regulated by factors such as microenvironmental pH [48], intracellular calcium levels [49, 50], hypoxia [51], and inflammatory conditions [52]. Exosomes transported via the bloodstream are promptly cleared by phagocytes in the splenic marginal zone, liver Kupffer cells, dendritic cells (DCs), and macrophages in the lungs [37]. This remains an obstacle to be addressed in the therapeutic application of exosomes for targeting focal tissues in clinical practice (Fig. 2).

EVs, especially exosomes, have emerged as key mediators of intercellular communication and promising therapeutic agents for wound repair [53]. Recent advances in exosome research have revealed that exosomes from diverse biological sources, such as stem cells, non-stem cells, and plants, exhibit distinct characteristics that influence their potential in wound healing applications. Stem cell-derived exosomes, particularly those from adipose tissue, bone marrow, and umbilical cord, are rich in regenerative bioactive molecules and demonstrate strong targeting abilities, low immunogenicity, and favorable biocompatibility, making them highly promising for therapeutic use [34]. Non-stem cell exosomes, such as those derived from keratinocytes or body fluids like milk and serum, share many molecular biomarkers with stem cell exosomes but show greater variability in immunogenicity and biosafety, depending on their origin and purification method. Meanwhile, plant-derived exosomes offer an emerging alternative, featuring exceptional biocompatibility, oral bioavailability, and scalability at low cost, though they face challenges in drug loading and standardization due to species-specific variability [54, 55]. In conclusion, the identified differences and characteristics suggest that the selection of exosomes should be guided by therapeutic objectives, production feasibility, and safety considerations (Table 1).

**Table 1:** Exosomes from different origins in promoting wound healing

Catalogues	Stem cell-derived exosomes	Non-stem cell exosomes	Plant-derived exosomes
Primary sources	Adipose tissue, bone marrow, placentas (human amniotic, human umbilical cord, et al.), menstrual blood, epidermis, and hair follicles, et al.	Skin cells (keratinocytes, fibroblasts, et al.), body fluids (serum, saliva, milk, cerebrospinal fluid, urine, semen, et al.).	Roots, leaves, fruits, and seeds from various plants, such as fruits, vegetables, spices, medicinal plants, et al.
Size range (nm)	30–150	30–150	50-1000
Specific biomarkers	CD63, CD81, CD9, HSP60, HSP70, HSP90, ESCRT complex, ALIX, TSG 101, flotillin, et al.	CD63, CD81, CD9, HSP60, HSP70, HSP90, ESCRT complex, ALIX, TSG 101, flotillin, et al.	PEN1, PEN3, Tetraspanin-8, HSP70, GAPDH, S-adenosyl-homocysteinase, et al.
Key bioactive cargoes	Specific molecules including lipids, proteins, nucleic acids, metabolites	Similar to stem cell-derived exosomes	Specific molecules including lipids, proteins, nucleic acids, metabolites, and other bioactive substances, such as vitamin C, ions, polysaccharides, oligosaccharides, polyphenols, flavonoids, carotenoids, et al.
Potential as drug delivery vehicles	Strong targeting, low immunogenicity, good biocompatibility, modifiable, mature loading/engineering.	Usable as carriers; immunogenicity and tumorigenicity vary; high-yield and oral potential in fluids like milk.	Excellent biocompatibility, stability, oral bioavailability; high yield, low cost; very low immunogenicity; and challenges in loading.
Biocompatibility and Immunogenicity	Lower immunogenicity, (esp. MSC); risks from donor variability and culture conditions.	Immunogenicity varies; pathogen and compatibility risks.	Extremely low immunogenicity; no human/zoonotic pathogens; safety dependent on plant source (e.g., phytotoxins and pesticide residues).
Production and Scalability	High cost and complexity; limited scalability; GMP challenges	Similar issues as SC-Exos; some fluids scalable (e.g., milk and colostrum) but difficult purification and heterologous proteins.	Low cost, high yield; scalable via plant cultivation; purification relatively mature; batch variability based on species/growth.

## Roles and mechanisms of ADSC-Exos in wound healing

ADSC-Exos contain a diverse array of bioactive substances, including metabolites, proteins, DNA, and ncRNAs [56], which regulate various aspects of the wound healing process, such as the inflammatory response, cell proliferation, and migration, angiogenesis, and collagen remodeling, thereby promoting tissue repair. Numerous studies have highlighted the significance of ADSC-Exos in wound healing, emphasizing their essential role in regulating various cellular processes and coordinating the release of growth factors [57].

The primary types of wounds include normal wounds, diabetic ulcers, burns, and pressure ulcers. These wounds are influenced by various stressors, such as hyperglycemia, thermal injury, and sustained mechanical stress, which creates distinct microenvironments that impact wound healing through mechanisms like chronic inflammation, redox imbalance, and impaired angiogenesis. Preclinical evidence indicates that ADSC-Exos exhibit adaptive therapy within these microenvironments by targeting and modulating these pathological cascades. In the case of normal full-thickness wounds, ECM@exo, an ECM hydrogel containing ADSC-Exos, achieved a wound closure rate of 96.4% ± 0.9% by day 14, significantly outperforming the drug control group (74.5% ± 3.9%) and the ECM hydrogel alone (82.4% ± 2.0%) [58]. Mechanistically, ECM@exo enhanced proliferative activity, increasing Ki67 + cell levels by 3.2-fold and proliferating cell nuclear antigen (PCNA) expression by 2.8-fold compared to controls, demonstrating its synergistic effect in accelerating intrinsic healing processes. In diabetic models, however, additional challenges were observed. Untreated wounds showed less than 50% closure, while ECM@exo facilitated healing to 92%, promoting angiogenesis and epidermal regeneration, as reported by Ren et al. Burn injuries, on the other hand, necessitate combinatorial biomaterial strategies. Cross-linking ADSC-Exos with chitosan-αβ-glycerophosphate hydrogels accelerated healing, reducing inflammation and promoting epithelial migration [59]. Diabetic wounds are often complicated by poor glycemic control, burn wounds by tissue damage and infection, and pressure ulcers by persistent pressure. ADSC-Exos may address these different wound types through various mechanisms. However, to date, no studies have directly compared the efficacy of ADSC-Exos across these distinct wound applications. Recent research typically focuses on their effectiveness in either single or multiple models, emphasizing the need for clinical studies and cross-sectional comparisons of ADSC-Exos across different wound types as key areas for future research.

### Immune regulation

During the inflammatory phase of wound healing, vasodilation, and increased capillary permeability facilitate the recruitment of various immune cells from the bone marrow to the wound site, enabling the clearance of cellular debris, pathogenic microorganisms, and apoptotic cells in preparation for the proliferative phase [60]. Neutrophils eliminate microbial pathogens, including bacteria and fungi, through a ROS-dependent manner and secrete chemokines to attract additional immune cells to the wound area [61]. ROS, as oxygen-derived chemical mediators, play a key role in modulating oxidative stress and inflammatory responses within the wound microenvironment [62]. Macrophages exhibit dual functionality during wound healing by dynamically polarizing into M1 (pro-inflammatory) and M2 (pro-reparative) phenotypes, thereby coordinating different phases of tissue repair. In the early stage of inflammation, M1 macrophage activation is initiated by TLR2/4 recognition of pathogen- and damage-associated molecular patterns, such as microbial lipopolysaccharides and extracellular nucleotides [63]. This polarization is further promoted by Th1-derived cytokines, including IFN-γ and TNF-α. M1 macrophages execute bactericidal functions through phagocytosis of microorganisms and necrotic debris while producing cytotoxic oxidants such as superoxide radicals and peroxynitrite precursors, along with pro-inflammatory cytokines (IL-6, IL-12) and chemokines that recruit adaptive immune cells [64]. These responses enhance innate immunity via activation of the NF-κB/STAT1/5 signaling pathway, thereby ensuring microbial clearance and priming of the wound microenvironment [65]. CD80/86 and MHC II are commonly recognized surface markers of M1 macrophages. During the proliferative phase of wound healing, M2 macrophage polarization is induced by Th2-associated cytokines, including IL-4, IL-13, and IL-10, which collectively suppress inflammation and promote tissue regeneration [66]. M2 macrophages reduce the production of ROS and downregulate pro-inflammatory cytokines such as TNF-α, while simultaneously secreting anti-inflammatory mediators like IL-10 and TGF-β, as well as regenerative growth factors including VEGF, PDGF, and epidermal growth factor (EGF) [67]. These molecular cues enhance collagen synthesis by fibroblasts, stimulate angiogenesis, facilitate the recruitment of stem and progenitor cells through chemokines such as CCL17 and CCL24, and contribute to ECM remodeling. M2 macrophage activity is further marked by high expression of surface receptors CD206 and CD163 and by chemotactic signaling mediated through CCL17, CCL18, CCL22, and CCL24 pathways [68]. A timely transition from M1 to M2 macrophages is critical for normal wound healing: M1 macrophages clear pathogens and prepare the wound bed, while M2 macrophages drive structural repair by resolving inflammation and coordinating stromal regeneration [69]. Disruption of this polarization balance can lead to pathological conditions such as chronic wounds or fibrosis, highlighting the therapeutic potential of targeting macrophage plasticity.

The inflammatory response is a natural stage in the wound-healing process and serves as a self-defense mechanism for the body. However, chronic and excessive inflammation can lead to delayed wound healing. Modulating the immune response to promote the transition from the inflammatory to the proliferative phase can accelerate the wound-healing process.

Recent studies have shed light on the role of ADSC-Exos in modulating inflammation via ncRNAs. For example, circRps5, a circRNA found in ADSC-Exos, promoted M2 macrophage polarization by acting as a molecular sponge for miR-124-3p, which otherwise inhibited DUSP1 expression [70]. A deficiency in DUSP1 led to heightened activation of the MAPK pathway, thereby promoting M1 polarization and increasing the production of pro-inflammatory cytokines such as IL-6 and TNF-α. By sequestering miR-124-3p, circRps5 restored DUSP1-mediated MAPK dephosphorylation, mitigating chronic inflammation in diabetic wounds. Additionally, Xu et al. demonstrated that ADSC-Exos enriched with miR-194 directly targeted the TGF-β1 promoter, suppressing TGF-β1-induced fibrosis in hypertrophic scar fibroblasts and reducing downstream inflammatory mediators, including IL-1β and IL-6 [71]. Another study found that let-7i-5p, delivered via ADSC-Exos, silenced GAS7 in keratinocytes under oxidative stress, thereby reactivating phosphatidylinositol 3-kinase (PI3K)/protein kinase B (AKT) signaling to promote cell survival and inhibit NF-κB-driven transcription of pro-inflammatory cytokines [45]. Collectively, these findings stressed the role of ADSC-Exo-derived ncRNAs as master regulators of inflammatory resolution through multi-target mechanisms. These regulatory pathways operate at various levels: miR-194 functions as a transcriptional repressor of TGF-β1; circRps5 acts post-transcriptionally to reprogram macrophage phenotypes; and let-7i-5p integrates pro-survival signaling with suppression of NF-κB activity. This multilayered regulatory strategy enables simultaneous control of inflammation initiation, progression, and tissue damage while minimizing compensatory cross-talk. Notably, the cell-specific action of ADSC-Exo-derived ncRNAs ensures spatially precise modulation of inflammation, reducing the risk of systemic off-target effects. Although these findings highlight the therapeutic promise of ADSC-Exos, optimizing ncRNA combinations to maximize synergistic anti-inflammatory effects remains a challenge. Rigorous validation is essential to avoid pathway saturation. Future research should utilize single-cell RNA sequencing and spatial transcriptomics to evaluate ncRNA delivery efficiency in vivo. In addition, preclinical studies must determine appropriate dosage thresholds to balance therapeutic efficacy with potential toxicity.

In vitro, ADSC-Exos have been shown to reduce apoptosis and enhance the phagocytic activity of neutrophils, thereby promoting more efficient pathogen clearance [72]. In injured tissues, elevated secretion of interferon-α (IFN-α) by T cells contributes to the accumulation of pro-inflammatory M1 macrophages and the persistence of inflammation [73]. Blazquez et al. demonstrated that ADSC-Exos could inhibit T-cell activation and reduce IFN-α secretion in vitro, highlighting their anti-inflammatory potential [74]. Moreover, ADSC-Exos promoted the transition of macrophages from the pro-inflammatory M1 phenotype to the pro-repair M2 phenotype. Kouroupis et al. demonstrated that ADSC-Exos promoted M2 polarization by upregulating anti-inflammatory mediators such as IL-10 and arginase-1 (Arg-1) and inhibiting pro-inflammatory cytokines like TNF-α and IL-6 [75]. Liebmann et al. reported that macrophages stimulated by aCGRP IFP-MSC sEVs switched to the M2 phenotype [76], which aligns with our findings on ADSC-Exos-mediated anti-inflammatory polarization of macrophages. In a wound inflammation model induced by IFN-γ and TNF-α, cells treated with ADSC-Exos showed notable differences in the expression of anti-inflammatory and wound healing-related miRNAs compared to the control group. MiRNAs, small ncRNAs approximately 22 nucleotides long, regulate wound repair processes by binding to target mRNAs and modulating gene expression post-transcriptionally [77].

Song et al. investigated the immunomodulatory effects of ADSC-Exos in both healthy and diabetic mouse wound healing models [58]. In normal wound models, ADSC-Exos attenuated the inflammatory response by downregulating pro-inflammatory cytokines TNF-α and IL-6 and promoting M2 macrophage polarization. These changes contributed to enhanced wound healing, increased collagen deposition, and stimulated cell proliferation. Xiao et al. developed a human acellular amniotic membrane (hAAM) scaffold loaded with ADSC-Exos in vitro and transplanted it into diabetic mouse wounds [78]. The hAAM-Exos group exhibited the lowest number of inflammatory cells, along with enhanced M2 macrophage recruitment, indicating that ADSC-Exos effectively suppressed wound inflammation. Moreover, wounds treated with ADSC-Exos demonstrated improved healing outcomes and superior new skin formation compared to controls. In another study, Yin et al. cultured ADSCs transfected with a circRps5-overexpressing plasmid (e-circRps5) under hypoxic conditions and injected the derived exosomes into the wound margins of diabetic mice [70]. Compared to controls, treatment with these ADSC-Exos significantly reduced infiltration of inflammatory cells such as lymphocytes and neutrophils and lowered levels of C-reactive protein (CRP), thereby alleviating chronic inflammation in diabetic wounds.

ADSC-Exos can also modulate key signaling molecules in inflammatory pathways by delivering long non-coding RNAs (lncRNAs), thereby influencing the biological behavior of downstream immune cells [79]. LncRNAs, defined as non-protein-coding transcripts longer than 200 nucleotides, regulate chromatin structure and transcription by recruiting chromatin-modifying complexes, mechanisms that are crucial for tissue regeneration and repair [80]. Li et al. found that ADSC-Exos delivered lncRNA H19, which targets miR-130b-3p, thereby promoting macrophage polarization toward the M2 phenotype. This polarization, in turn, enhanced fibroblast proliferation and migration, as well as angiogenesis [81]. Additionally, ADSC-Exos significantly promoted the release of anti-inflammatory factors associated with M2 macrophages by upregulating the expression of Rho-associated coiled-coil containing protein kinase 1 (ROCK1) and phosphatase and tensin homolog deleted on chromosome ten (PTEN) [82]. In a lipopolysaccharide (LPS)-induced inflammatory model using human dermal fibroblasts (HDFs), Patel et al. reported that GAS5, a key lncRNA involved in vitro wound repair, was highly enriched in ADSC-Exos. They further demonstrated that these exosomes modulated the expression of toll-like receptor 7 (TLR7) and other related targets in a GAS5-dependent manner, thereby influencing inflammatory pathways [83].

### Cell proliferation and re-epithelialization

During the proliferative phase of wound healing, fibroblasts play a critical role by producing collagen, promoting ECM deposition, and transforming the wound microenvironment from an inflammatory to a regenerative state [84]. These fibroblasts can differentiate into myofibroblasts, contributing to wound contraction and closure. Simultaneously, keratinocytes proliferate and migrate toward the wound center to aid in re-epithelialization. Therefore, promoting the proliferation and migration of fibroblasts and keratinocytes is essential for accelerating ECM synthesis in injured tissues, thereby enhancing the overall healing process. Numerous studies have demonstrated that ADSC-Exos significantly improved the viability of both fibroblasts and keratinocytes. ADSC-Exos can be internalized by fibroblasts, inducing their proliferation, migration, collagen production, and the expression of genes such as N-cadherin, cyclin-1, and PCNA in a dose-dependent manner [85]. Additionally, systemic administration of ADSC-Exos has been shown to increase type I and type III collagen synthesis during the early healing stages, while reducing type I collagen expression in later stages, thereby minimizing scar formation. Zhang et al. further demonstrated that ADSC-Exos promoted collagen deposition both in vivo and in vitro, potentially via activation of the phosphorylated PI3K/AKT signaling pathway [86]. Moreover, ADSC-Exos enhanced the proliferation and migration of HaCaT keratinocytes in vitro by upregulating AKT phosphorylation and HIF-1α expression and accelerated wound closure in a full-thickness mouse wound model through activation of the AKT/HIF-1α pathway [87].

In diabetic wounds, the hyperglycemic environment impairs fibroblast and keratinocyte proliferation and disrupts the re-epithelialization process [88]. Hsu et al. isolated ADSCs from diabetic mice extracted their exosomes, and demonstrated that ADSC-Exos from diabetic mice could activate the TGF-β/Smad3 signaling pathway, thereby promoting collagen synthesis and fibroblast activation to facilitate diabetic wound healing [89]. These findings suggested that ADSC-Exos could enhance cell proliferation and re-epithelialization in both diabetic and healthy conditions.

### Angiogenesis

Neovascularization is crucial for effective wound healing, as it supplies the necessary materials to support fibroblast and epidermal cell proliferation and tissue regeneration. The process of neovascularisation involves the activation of endothelial cells in local microvessels [90]. Under hypoxic conditions, vascular endothelial cells respond to various hypoxia-inducible growth factors, become activated, and begin degrading the ECM in granulation tissue. They then proliferate, migrate, and sprout outward to form new capillaries [91]. Enhancing the release of hypoxia-responsive growth factors and stimulating endothelial cell activity can thus promote neovascularization in the injured area.

ADSC-Exos can enhance angiogenesis during wound healing by promoting the proliferation and migration of vascular endothelial cells through the delivery of their bioactive contents [92]. They are enriched with several pro-angiogenic miRNAs, including miR-132, miR-146a, and miR-125a [93, 94]. Among these, miR-132 and miR-146a derived from ADSC-Exos promoted angiogenesis in endothelial cells by inhibiting the expression of anti-angiogenic genes thrombospondin-1 (*THBS1*) and Vasohibin-1 (*VASH1*), respectively [82]. In addition, ADSC-Exos could deliver miR-125a to endothelial cells, targeting and suppressing the angiogenesis inhibitor Delta-like 4 (DLL4), thereby increasing the population of CD34⁺ vascular endothelial tip cells [95]. ADSC-Exos also facilitate revascularization through hypoxia-sensitive ncRNA circuits. Li et al. identified that hypoxic preconditioning enriches lncRNA H19 in ADSC-Exos, which stabilizes HIF-1α by recruiting USP22 for deubiquitination [44]. Stabilized HIF-1α enhanced VEGFA transcription in endothelial cells, thereby promoting capillary formation in diabetic wounds. Similarly, miR-146a-5p in ADSC-Exos targets JAZF1, relieving its inhibitory effect on STAT3 phosphorylation [96]. Activated STAT3 subsequently upregulates VEGFA and ANGPT2, promoting endothelial cell proliferation and ECM remodeling. Furthermore, Wang et al. found that hypoxia-induced circ-0001747 in ADSC-Exos functions as a sponge for miR-199a-5p, thereby de-repressing HIF-1α and establishing a feedforward loop that sustains PDGFB- and ANGPT2-mediated angiogenesis [97]. These findings stressed the ability of ADSC-Exos to adapt to the hypoxic wound microenvironment and the critical role of their derived ncRNAs in orchestrating the activation of vascular endothelial cells during tissue repair.

In diabetic wounds, the high-glucose environment induces excessive production of ROS, leading to mitochondrial dysfunction, apoptosis, and inflammation in vascular endothelial cells. These effects collectively impair vascular function and delay the healing of diabetic wounds [98]. Zhang et al. demonstrated that ADSC-Exos reduced ROS levels in vascular endothelial cells by upregulating the expression of sirtuin 3 (SIRT3) and superoxide dismutase 2 (SOD2), thereby preserving mitochondrial function and promoting diabetic wound healing [99].

### Collagen remodeling

The tissue remodeling phase involves the regression of neovascularization, the synthesis of matrix metalloproteinases (MMPs) by myofibroblasts for the targeted degradation of specific ECM components, and the subsequent transformation of granulation tissue into scar tissue [100]. MMPs, which are zinc-dependent endopeptidases, degrade ECM constituents to promote tissue remodeling and facilitate cell migration [101]. Keloid hyperplasia is a histopathological alteration of the skin following wound healing; excessive keloid formation can compromise skin aesthetics and impair its functional integrity [102].

ADSC-Exos are enriched with various anti-fibrotic bioactive components that effectively regulate collagen remodeling and inhibit keloid hyperplasia. ADSC-Exos inhibit the proliferation, migration, collagen deposition, and differentiation of proliferative human scar-derived fibroblasts (HSFs) into myofibroblasts [103]. This effect was mediated by miR-192-5p carried by ADSC-Exos, which targets IL-17RA and inhibits the Smad pathway involved in proliferative scar fibrosis. Consequently, ADSC-Exos reduced collagen deposition during the late stage of wound healing, as demonstrated in a mouse model of full-thickness skin defects. MiR-181a, known to be associated with tissue fibrosis, was highly expressed in proliferative scar tissue [104]. Chen et al. reported that ADSC-Exos attenuated collagen deposition and α-smooth muscle actin (α-SMA) production in HSFs by downregulating miR-181a and upregulating SIRT1 expression [105]. α-SMA, a contractile protein, is a key biomarker of activated myofibroblasts involved in tissue contraction and scarring [106]. Moreover, ADSC-Exos promote ECM remodeling by regulating MMP expression. Intravenous administration of ADSC-Exos increased the ratios of type III collagen to type I collagen, TGF-β3 to TGF-β1, and MMP3 to TIMP1, thereby reducing scar size in mice [107]. Additionally, exosome therapy was found to inhibit fibroblast-to-myofibroblast transformation and modulate ECM remodeling via activation of the ERK/MAPK pathway [108].

However, it has also been suggested that ADSC-Exos could promote collagen deposition in fibroblasts during the later stages of wound healing, potentially contributing to scar formation. For instance, in a study by Wang et al. on diabetic wounds, ADSC-Exos treatment enhanced collagen deposition in injured tissues during the late healing phase [109]. These conflicting findings may be attributed to the dynamic nature of signaling between ADSC-Exos and fibroblasts, as well as the stage-specific regulatory effects of ADSC-Exos on ECM synthesis. Therefore, further in-depth exploration is still needed regarding the effect of ADSC-Exos on collagen production by fibroblasts and its relationship with scar proliferation.

## Engineering strategies for boosting the efficacy of ADSC-Exos in wound healing

This section discusses the application of ADSC-Exos in wound healing, initially highlighting the regenerative potential of natural ADSC-Exos. ADSC-Exos promote wound healing through various molecular mechanisms, including the regulation of miRNAs and lncRNAs. Natural ADSC-Exos facilitate wound repair by modulating key biological processes such as autophagy, cell proliferation, and migration, and they offer advantages such as high biocompatibility and a lower risk of immune rejection. However, their therapeutic efficacy may be influenced by the extraction method and individual variability, leading to less controllable activity and composition, as well as potential batch-to-batch inconsistencies in clinical applications.

Exosome gene editing involves the use of genetic engineering techniques, such as CRISPR/Cas9, to modify the genetic material of ADSCs, thereby enhancing or introducing specific functions in the exosomes they produce. This approach enables the precise loading of therapeutic molecules, including miRNAs and proteins, to target specific pathological processes, providing a more tailored strategy for disease treatment. However, gene editing poses concerns regarding off-target effects that may disrupt unintended cellular pathways, as well as ethical considerations associated with permanent genetic modifications. Despite its promising specificity and efficacy, the high cost and technical complexity of this method currently limit its broad clinical application.

Pre-processing of ADSCs involves exposing the cells to pharmacological agents or environmental conditions, such as hypoxia, to transiently enhance the therapeutic properties of their exosomes. This treatment can increase the yield of exosomes enriched with beneficial proteins and RNAs, thereby enhancing their regenerative potential. However, the effects of such preconditioning are often temporary and may vary significantly across different cell batches, resulting in inconsistencies in exosome quality and function. Additionally, the lack of long-term stability in the induced changes presents challenges for clinical scalability and reproducibility.

**Fig. 3:**
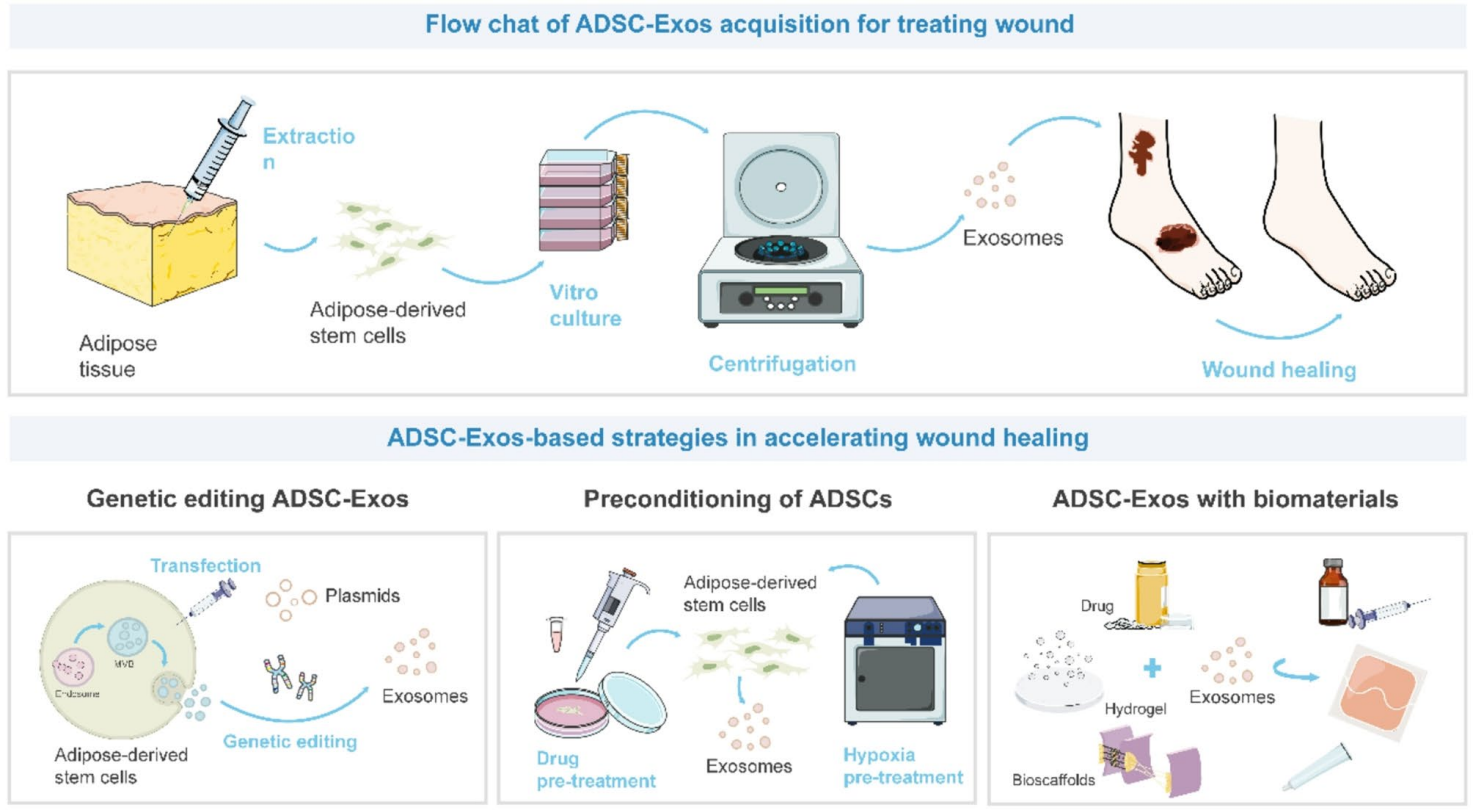
Applications of ADSC-Exos in wound healing. Autologous adipose-derived stem cells (ADSCs) can be largely isolated human adipose tissue through liposuction, subsequently purified and cultured in vitro. ADSC-Exos are frequently obtained from culture medium supernatants by ultracentrifugation and possess extraordinary tissue repair and wound healing capabilities. Current potentiation strategies of ADSC-Exos for accelerating wound healing mainly include gene editing of ADSC-Exos, preconditioning of ADSCs, and ADSC-Exos with biomaterials

Combining exosomes with biocompatible materials, such as liposomes or hydrogels, improves the stability, delivery efficiency, and targeted release of therapeutic exosomes. This approach helps protect exosomes from premature degradation in the body and enables controlled release at the target site, which is essential for applications in chronic wound healing and tissue regeneration. However, challenges such as potential immunogenicity and batch-to-batch variability in material properties, may affect the consistency and safety of the final therapeutic product (Fig. 3).

### Natural ADSC-Exos

The prospective function of natural ADSCs and ADSC-Exos in tissue repair has been confirmed by a multitude of studies. An extensive investigation has been conducted by researchers into the molecular mechanisms that regulate this regenerative process. Mouse keratinocytes were treated with ADSC-Exos by An et al., and significant upregulation of pathways essential for wound healing was observed. These pathways encompass responses to viruses, bacteria, immune reactions, and tissue injuries [110]. Analysis of endogenous RNA networks unveiled that Neat1 induced Ulk1 expression, triggering autophagy by sponging miR-17-5p, thus markedly enhancing wound healing. Findings suggested that lncRNA Neat1 carried by ADSC Exos could serve as a promising target for challenging skin wound treatments. This study provided novel insights into the implementation of ADSC-Exos in skin wound management and advanced the use of ADSC-Exos therapy in clinical practice. LncRNA MALAT1 from human ADSC-Exos facilitated the proliferation and migration of HSFs and expedited the healing of skin wounds by inhibiting miR-378a, thereby increasing the expression of FGF2 [111]. To identify the substantially differentially expressed lncRNAs in mouse skin tissues following treatment with ADSC-Exos, Zhu et al. employed microarray analysis. ADSC-Exos restored the mRNA expression of discoidin domain receptor 2 (DDR2) by delivering X-inactive-specific transcript (XIST) to silence miR-96-5p. DDR2, a collagen-binding tyrosine kinase, mediates fibroblast-ECM interactions to activate pro-fibrotic pathways [112]. XIST, a lncRNA encoded within the X chromosome inactivation center, plays a role in fibroblast activation and ECM synthesis after injury and is also implicated in oncogenic pathways [9]. Its involvement in burn wound repair further supports its therapeutic potential [113]. This mechanism enhanced the proliferation and migration of mouse dermal fibroblasts (MDFs), reduced inflammatory infiltration, increased collagen deposition in trabecular tissue, and accelerated wound healing in mice [114].

### Genetic editing ADSC-Exos

Despite the promising potential of natural ADSC-Exos in promoting wound healing, their clinical application still encounters several challenges, including low concentrations of active components, short half-life, and rapid degradation at the wound site, all of which hinder the effectiveness and sustainability of wound therapy [56]. To address these limitations, engineered exosomes have been extensively investigated as promising candidates for novel exosome-based nanotherapies [115]. Exosome engineering strategies are generally categorized into direct and indirect modifications [116]. Direct modifications involve the creation of exosome mimics through techniques such as electroporation, sonication, freeze-thaw cycles, and cargo loading, or the use of biomimetic molecules including exosomal proteins and lipids. Indirect modifications include gene editing or pretreatment of parental cells to alter the bioactive contents of the exosomes. Current approaches to engineering ADSC-Exos for wound healing primarily focus on gene modification of ADSCs, pretreatment of ADSCs, and the integration of exosomes with functional materials.

Genetic engineering of ADSCs provides a targeted approach to enhance the regenerative potential of ADSC-Exos by selectively enriching exosomal ncRNA content. Since exosomal ncRNAs reflect the molecular profile of their parental ADSCs, the overexpression of repair-associated ncRNAs in ADSCs directly increases their incorporation into exosomes, thereby improving therapeutic efficacy. For example, the strategic overexpression of miR-21 in ADSC-Exos leveraged its crucial role in wound healing by promoting keratinocyte proliferation, migration, survival, and suppressing apoptosis, thus facilitating re-epithelialization and tissue regeneration [117, 118]. Mechanistically, miR-21 inhibited TGF-β1 to attenuate SMAD2/3-mediated fibrotic signaling, while concurrently activating the PI3K/AKT/mTOR pathway via PTEN targeting, thereby enhancing cell cycle progression and resistance to apoptosis [119]. In addition, miR-21 contributed to ECM remodeling by downregulating fibroblast-inhibitory factors, promoting collagen synthesis, and preventing pathological fibrosis. This dual regulation of pro-regenerative and anti-fibrotic pathways positioned miR-21 as a key mediator of balanced skin repair, simultaneously resolving inflammation and accelerating tissue restoration. Yang et al. generated ADSC-Exos with elevated miR-21 expression by transfecting ADSCs with plasmids [120]. These engineered exosomes enhanced MMP-9 expression via the PI3K/AKT pathway, thereby promoting keratinocyte proliferation, migration, and wound closure. Similarly, miR-146a has been identified as a positive regulator of angiogenesis [121]. Che et al. reported that treatment of human umbilical vein endothelial cells (HUVECs) with ADSC-Exos resulted in elevated levels of miR-146a-5p, which enhanced the proliferation, migration, and angiogenic potential of HUVECs by targeting and inhibiting *JAZF1* [96]. Ge et al. further demonstrated that lentiviral transfection and ultracentrifugation-mediated enrichment of miR-132 in ADSC-Exos alleviated inflammation, enhanced angiogenesis, and promoted M2 macrophage polarization through the NF-κB signaling pathway [73]. In addition to miRNAs, lncRNAs, and circRNAs also play significant regulatory roles in wound healing [122]. CircRNAs, which are covalently closed RNA loops, function as miRNA sponges or transcriptional regulators within cellular signaling pathways [123]. Qiu et al. demonstrated that linc00511-overexpressing ADSC-Exos accelerated angiogenesis and promoted the healing of diabetic foot ulcers by inhibiting PAQR3-mediated ubiquitin degradation of Twist1 [124]. Similarly, ADSC-Exo-overexpressing mmu_circ_0001052 enhanced cell proliferation, migration, and angiogenesis in diabetic foot ulcers by suppressing miR-106a-5p expression and apoptosis, while activating the FGF4/p38MAPK signaling pathway [125]. Li et al. reported that NRF2-overexpressing ADSC-Exos more effectively promoted angiogenesis and reduced oxidative stress in diabetic wounds compared to control exosomes [126]. These findings suggested that genetically modifying protein expression in ADSCs was also an effective strategy to enhance the therapeutic potential of ADSC-Exos. In another study, Liebmann et al. investigated EVs derived from subpatellar fat pad mesenchymal stem cells (MSCs) that were genetically engineered to target calcitonin gene-related peptide (CGRP) and pain signaling pathways [76]. The genetically engineered sEVs CGRP while retaining their inherent ability to promote macrophage M2 polarization. This offered new insights into optimizing gene-edited ADSC-Exos for chronic wound therapy through the genetic design and production of multifunctional ADSC-Exos capable of simultaneously suppressing inflammation, inhibiting nociceptive signaling, and enhancing M2 macrophage-mediated tissue repair. These multifunctional ADSC-Exos target the triad of intractable pain, chronic inflammation, and delayed reepithelialization, which are the key factors contributing to the difficulty in treating refractory wounds.

Extensive research provides strong evidence that genetically modified ADSC-Exos represent a more effective therapeutic approach for chronic wound treatment. While genetic engineering enables the precise loading of therapeutic molecules with high specificity and efficacy, the associated technical complexity and biosafety concerns highlight the need for complementary strategies.

### Pre-processing of ADSCs

Unlike genetic editing, which is a highly targeted but resource-intensive method, pretreatment strategies use external stimuli such as pharmacological agents, cytokines, or physical factors to transiently activate ADSCs before exosome collection. This approach leverages the adaptability of stem cells to their environment and avoids permanent genomic changes, making it technically simpler, safer, and more broadly applicable than genetic engineering.

The secretion of exosomes is closely influenced by the physiological state of the parental cells; thus, pretreatment of ADSCs inevitably alters the composition of ADSC-Exos [35, 127]. Several studies have demonstrated that pre-processing MSCs with pharmacological agents, cytokines, or physical stimuli can enhance the regenerative potential of both MSCs and their derived exosomes [128–135]. Compared to genetically engineered exosomes, this pretreatment strategy is technically simpler, safer, and more commonly employed.

#### Drug pre-treatment

The preconditioning of ADSCs with drugs can enhance the biological activity and regenerative function of ADSC-Exos. Selenium is a well-known antioxidant and cofactor for various enzymes that inhibit oxidative stress and inflammation. Heo et al. pretreated ADSCs with selenium and collected exosomes (Sei-ADSC-Exos), further demonstrating that Sei-ADSC-Exos exhibited enhanced antioxidant, anti-inflammatory, and pro-regenerative properties compared to control exosomes. Greater potential in promoting wound healing was verified by HDFs cultured in vitro and wound healing in mice in vivo [136]. Wu et al. used LPS to stimulate ADSCs and collected LPS-ADSC-Exos for the treatment of vascular endothelial cells [137]. The results showed that LPS-ADSC-Exos significantly promoted the migration and angiogenesis of vascular endothelial cells compared to control exosomes. Proteomic analysis revealed that several angiogenesis-related proteins, such as histone deacetylase (HDAC), amyloid beta A4 protein (APP), and integrin beta-1 (ITGB1), were highly expressed in LPS-ADSC-Exos.

#### Hypoxia pre-treatment

Oxygen tension is widely recognized as a key factor influencing the biological behavior of MSCs in culture [138]. Studies have shown that hypoxic preconditioning of ADSCs induces alterations in the protein and miRNA profiles of their exosomes, thereby affecting the therapeutic efficacy of ADSC-Exos in skin wound treatment. Wang et al. reported that several wound healing-related miRNAs, such as miR-21-3p, miR-126-5p, and miR-31-5p, were significantly upregulated in hypoxia-conditioned ADSC-Exos (Hpy-ADSC-Exos) compared to normoxic controls [139]. These Hpy-ADSC-Exos enhanced diabetic wound healing by activating the PI3K/AKT signaling pathway and exhibited superior therapeutic efficacy relative to control exosomes [140]. In another study, Shi et al. demonstrated that hypoxia treatment increased the expression of circ-Snhg11 in ADSC-Exos, which promoted macrophage polarization toward a pro-repair phenotype. This was achieved by delivering circ-Snhg11 to target the miR-144-3p/HIF-1α axis, ultimately facilitating the healing of diabetic foot ulcers [141].

### Combination ADSC-Exos with biomaterials

Despite their excellent tissue regeneration-promoting properties, the limited therapeutic efficacy of ADSC-Exos in wound healing remains a major challenge. ADSC-Exos are typically administered via injection, which leads to their rapid clearance from circulation and a short duration of action at the target site [142]. To address this, recent research has focused on combining exosomes with biomaterials that can prolong their retention on wound surfaces without compromising biological activity [143]. Hydrogels, as highly hydrophilic biomaterials, are capable of preserving the bioactivity of cell secretions while allowing for the sustained release of active therapeutic agents [144]. Hydrogels significantly improve the therapeutic performance of ADSC-Exos by overcoming limitations related to stability, controlled release, and retention. The three-dimensional porous network of hydrogels enables regulation of exosome release kinetics by adjusting pore size relative to exosome diameter, effectively preventing burst release and extending retention during degradation [145]. Electrostatic interactions between cationic hydrogels, exemplified by chitosan, and the anionic phospholipid membranes of exosomes enhanced drug-loading efficiency and reduced premature clearance [146]. Furthermore, the incorporation of ECM-mimetic adhesion peptides, such as RGD and DGEA, or fusion peptides with collagen-binding domains, including CP05-conjugated variants, reinforces matrix-exosome integration and prolongs bioactivity at wound sites [147]. The addition of nanoclay enhanced the mechanical strength of hydrogels and reduced porosity [148], while also extending exosome retention through electrostatic immobilization. This sustained release aligned with the multi-phase nature of tissue repair, thereby maximizing therapeutic outcomes. For example, delayed exosome release from hydrogels supported sustained M1-to-M2 macrophage polarization, yielding significantly better results compared to unencapsulated exosomes [149]. In summary, hydrogels serve as dynamic reservoirs that stabilize ADSC-Exos, extend their local bioavailability, and synchronize their release with the temporal demands of tissue regeneration.

Notably, Yang et al. incorporated ADSC-Exos into a Pluronic F-127 hydrogel and demonstrated that this system enabled the sustained release of exosomes at the site of injury [150]. The Pluronic F-127/ADSC-Exos complex promoted wound healing by enhancing collagen regeneration and reducing inflammation. In addition to Pluronic F-127, GelMA hydrogels [151], β-chitosan nanofibre hydrogels [152], chitosan hydrogels [153], alginate hydrogels [154], and ECM hydrogels [58] have been shown to effectively load ADSC-Exos and improve their therapeutic efficacy in traumatic wounds. Moreover, specific modifications to hydrogels can endow hydrogel/exosome composites with unique biological functions. For instance, Wang et al. encapsulated ADSC-Exos in an injectable, thermosensitive, adhesive, and multifunctional polysaccharide-based hydrogel (FEP), enabling sustained pH-responsive exosome release [155]. This hydrogel exhibited a range of beneficial properties, including tissue adhesion, rapid hemostasis, strong antimicrobial activity, UV shielding, and self-healing. In a diabetic wound model, the FEP/ADSC-Exos dressing significantly accelerated wound healing by promoting cell proliferation, granulation tissue formation, angiogenesis, collagen remodeling, and re-epithelialization. Furthermore, the co-delivery of ADSC-Exos with therapeutic agents via hydrogels has emerged as a promising approach for wound treatment. Zhang et al. developed a dual-loaded hydrogel with adhesive, antioxidant, self-healing, and electrically conductive properties, designed to co-deliver ADSC-Exos and metformin [156]. This composite system reduced cellular ROS levels by inhibiting mitochondrial fission, thereby preserving F-actin homeostasis, alleviating microvascular dysfunction in a high-glucose environment, and effectively enhancing diabetic wound healing.

In addition to hydrogels, bioscaffolds are also widely employed for exosome delivery. Shiekh et al. embedded ADSC-Exos into a multifunctional bioscaffold possessing antioxidant, antimicrobial, and oxygen-releasing properties [157]. This bioscaffold acted synergistically with ADSC-Exos to reduce oxidative stress, stimulate angiogenesis, enhance collagen remodeling, and significantly accelerate the healing of diabetic wounds infected with Staphylococcus aureus and Pseudomonas aeruginosa. Similarly, Khalatbary et al. utilized a bioengineered microporous three-dimensional amniotic membrane scaffold (AMS) loaded with ADSC-Exos and demonstrated that the combined implantation significantly promoted angiogenesis and improved diabetic wound healing [158]. These findings further support the potential clinical application of combining ADSC-Exos with biomaterials in wound treatment (Table 2).

**Table 2:** Summary of preclinical applications of ADSC-Exos in wound healing

Application Type	ADSC-Exos Type/Modification	Key Mechanisms	Primary Outcomes	References
Natural ADSC-Exos	Unmodified ADSC-Exos	LncRNA Neat1 induces autophagy via sponging miR-17-5p to activate Ulk1	Significantly enhanced skin wound healing in mice	[110]
	Unmodified ADSC-Exos	LncRNA MALAT1 upregulates FGF2 by inhibiting miR-378a	Promoted proliferation/migration of HSFs and accelerated wound closure	[111]
	Unmodified ADSC-Exos	LncRNA XIST restores DDR2 expression via silencing miR-96-5p	Enhanced MDF proliferation, reduced inflammation, and collagen deposition	[114]
Genetically Engineered ADSC-Exos	miR-21-overexpressed ADSC-Exos	Upregulated MMP-9 through PI3K/AKT pathway	Accelerated keratinocyte proliferation/migration and wound healing	[120]
	miR-146a-overexpressed ADSC-Exos	Upregulated SERPINH1 and p-ERK	Enhanced fibroblast proliferation, migration, and neovascularization	[121]
	miR-132-overexpressed ADSC-Exos	Modulated M2 macrophage polarization via NF-κB pathway	Reduced inflammation and improved angiogenesis	[159]
	linc00511-overexpressed ADSC-Exos	Inhibited PAQR3-mediated Twist1 ubiquitination	Accelerated angiogenesis in diabetic foot ulcers (DFUs)	[76]
	mmu_circ_0001052-overexpressed ADSC-Exos	Activated FGF4/p38MAPK pathway by suppressing miR-106a-5p	Promoted cell proliferation/migration and suppressed apoptosis in DFUs	[77]
	NRF2-overexpressed ADSC-Exos	Enhanced antioxidant capacity	Improved angiogenesis and reduced oxidative stress in diabetic wounds	[78]
Preconditioned ADSCs	Selenium-treated ADSC-Exos	Enhanced antioxidant/anti-inflammatory properties	Superior wound healing via oxidative stress reduction and tissue regeneration	[89]
	LPS-stimulated ADSC-Exos	Enriched angiogenesis-related proteins (HDAC, APP, ITGB1)	Promoted endothelial cell migration and angiogenesis	[90]
	Hypoxia-preconditioned ADSC-Exos	Activated PI3K/AKT via upregulated miR-21-3p/miR-126-5p/miR-31-5p	Enhanced diabetic wound healing efficacy	[95]
	Hypoxia-preconditioned ADSC-Exos	Delivered circ-Snhg11 to regulate miR-144-3p/HIF-1α axis	Promoted macrophage pro-repair phenotype and DFU healing	[96]
Biomaterial-Combined ADSC-Exos	Pluronic F-127 hydrogel	Sustained exosome release	Improved collagen regeneration and anti-inflammatory effects	[100]
	FEP multifunctional hydrogel	pH-responsive release with antimicrobial/adhesive properties	Accelerated diabetic wound closure via angiogenesis and re-epithelialization	[106]
	Conductive hydrogel + metformin	Reduced ROS by inhibiting mitochondrial fission	Restored F-actin homeostasis and microvascular function in diabetic wounds	[107]
	Antioxidant/antimicrobial scaffold	Synergistic oxidative stress reduction	Enhanced healing in *S. aureus*/*P. aeruginosa*-infected diabetic wounds	[108]
	Amniotic membrane scaffold (AMS)	Angiogenesis promotion	Accelerated diabetic wound healing	[109]

## Limitations and prospects

Although previous studies have extensively examined the roles of miRNAs, lncRNAs, and proteins in ADSC-Exos, other important ncRNAs, such as piRNAs and tnRNAs, remain largely unexplored. These ncRNAs are known to regulate gene silencing, repress transposable elements, and mediate epigenetic modifications, thereby playing critical roles in inflammation resolution and tissue remodeling. For instance, piRNAs have been implicated in oxidative stress responses in diabetic wounds [22]. Similarly, tnRNAs, which influence mRNA stability and translation, may contribute to fibroblast activation, but their presence and function in ADSC-Exos have not been identified. This limited scope constrains our understanding of the full therapeutic potential of exosomal cargoes and highlights the need to investigate these underrepresented molecules to uncover novel therapeutic mechanisms.

Advanced sequencing technologies provide powerful tools for identifying novel therapeutic targets within ADSC-Exos. While current studies primarily focus on single molecular categories, such as miRNAs or proteins, multi-omics approaches integrating transcriptomics, proteomics, and metabolomics remain rare. For example, lipidomic analysis could elucidate how exosomal sphingolipids influence macrophage polarization, while metabolomic profiling may uncover metabolites that promote angiogenesis [22]. Furthermore, deep sequencing and single-particle analysis can comprehensively profile underrepresented molecules, assess cargo heterogeneity, and identify specific exosome subpopulations enriched with therapeutic components. The integration of these datasets, complemented by machine learning, could uncover synergistic molecular combinations that enhance therapeutic efficacy. For instance, one study demonstrated the role of exosomal lipid rafts in enhancing miRNA delivery to endothelial cells, but such comprehensive analyses remain limited [160]. Prioritizing multi-omics characterization will advance our understanding of the complex regulatory mechanisms of ADSC-Exos and facilitate their clinical translation.

Exosomes represent a highly heterogeneous population of cell-derived membrane structures, and their cargo composition is highly influenced by the physiological state of the parent cell. However, the underlying mechanisms governing exosome biogenesis and cargo loading remain poorly understood. Current exosome engineering strategies are largely based on empirical approaches, with limited mechanistic insights. To improve therapeutic specificity, bioengineering strategies such as surface modification with RGD peptides and encapsulation in hyaluronic acid-based hydrogels have been employed. For example, RGD-functionalized ADSC-Exos demonstrated a 50% increase in targeting efficiency to diabetic wounds compared to unmodified exosomes, while also reducing off-target accumulation in vital organs [161]. Further investigation into the molecular mechanisms of exosome formation and cargo incorporation is essential to provide a theoretical foundation for the precise design and targeted modification of engineered ADSC-Exos. Additionally, longitudinal studies using fluorescence or radiolabeling to track exosome biodistribution are critical for establishing pharmacokinetic profiles and optimizing dosing regimens.

Despite promising preclinical results, the clinical translation of ADSC-Exos faces significant challenges, particularly in scalable production and standardized quality control. The process of obtaining and culturing ADSCs is labor- and material-intensive, with key factors such as cell source, isolation methods, culture conditions, and medium composition affecting the yield and quality of ADSC-Exos [162]. Traditional isolation methods, such as ultracentrifugation, are associated with low yields, high variability, and contamination with other EVs or impurities [163, 164]. Methodological heterogeneity, including variations in isolation techniques and inconsistent characterization, continues to impede reproducibility and clinical relevance. For example, variability in CD63/CD9 expression limits translational potential [165, 166]. Emerging technologies such as the ultrafast-isolation system (EXODUS) have significantly improved exosome recovery rates, achieving over 90% purity within hours and effectively removing impurities such as protein aggregates [167]. Furthermore, integrating 3D bioreactor systems with hypoxia preconditioning has been shown to increase ADSC-Exos production by up to threefold, while maintaining functional cargo integrity [168]. To ensure standardized quality control, multi-parametric characterization integrating proteomics and miRNA sequencing is essential. Machine learning algorithms are also being applied to predict exosome quality based on real-time bioprocessing parameters, minimizing human intervention and enhancing reproducibility [169].

The long-term safety and efficacy of ADSC-Exos remain inadequately studied, particularly in disease-specific contexts such as diabetic or chronic wounds. Existing toxicological data are primarily derived from acute or single-dose studies in healthy animal models, which fail to account for altered biodistribution and toxicity responses in pathological conditions [170]. Preclinical studies often use varying exosome concentrations without clear mechanistic justification, making clinical extrapolation challenging. Moreover, while preliminary evaluations report no acute irritation or toxicity, the long-term biodistribution, immunogenicity, and potential off-target effects of ADSC-Exos remain poorly characterized. Tissue-specific accumulation and adverse effects in metabolically heterogeneous populations, such as elderly or immunosuppressed individuals, require further investigation [171, 172]. Biomaterial-based delivery systems, such as hydrogels, can provide controlled local release and reduce rapid in vivo clearance, but their long-term safety and efficacy in humans remain unproven [173, 174]. Additionally, significant species differences between animal and human skin, such as the presence of the panniculus carnosus in mice, limit the translational potential of preclinical wound healing models [175]. Rigorous safety and pharmacokinetic assessments in chronic wound cohorts, using standardized dosing and biocompatible carriers, are essential for clinical translation [176, 177].

The absence of harmonized regulatory standards for exosome-based therapies poses a significant barrier to clinical approval. Regulatory agencies, such as the FDA and EMA, mandate comprehensive evidence of product characterization, safety, and efficacy following Good Manufacturing Practice (GMP) guidelines. The International Society for Extracellular Vesicles (ISEV) MISEV2023 framework provides a foundational roadmap for regulatory compliance, promoting standardized protocols for exosome isolation, characterization, and reporting [178]. However, preclinical data from physiologically relevant models, such as humanized mice or non-human primates, are increasingly required to evaluate immunogenicity and tumorigenic potential before initiating clinical trials. Ethical considerations, including transparent donor consent for ADSC sourcing and equitable access to therapies, must also be addressed. Recent guidelines emphasize the importance of ethical oversight committees to audit exosome production workflows and ensure compliance with biosafety standards [163]. International collaborations are crucial for aligning regulatory requirements and expediting global clinical translation.

Advancing ADSC-Exos into clinical practice requires collaborative innovation across scalable biomanufacturing, quality control, comprehensive safety and efficacy validation, and regulatory and ethical oversight. Future efforts should focus on developing GMP-compliant manufacturing platforms, conducting multicenter longitudinal safety studies, and fostering stakeholder engagement to address ethical considerations and commercial challenges. By addressing these limitations, ADSC-Exos can unlock their full therapeutic potential in wound healing and beyond.

Clinical studies in recent years have shown that exosomes/EVs have the potential to treat various types of wounds with a favorable safety profile and efficacy. In a phase I trial, platelet-derived EVs (pEVs) showed excellent tolerability in wounds of healthy volunteers, and subcutaneous administration showed no significant adverse effects [179]. Notably, multiple sources of exosomes/EVs have shown significant results in complex refractory wounds. Pumford et al. topical application of pEVs achieved 96–100% closure of poorly healing scalp wounds following chemoradiation and surgery [180]. Bone marrow MSC-EVs (ExoFlo^®^) promote complete healing of recurrent pressure ulcers within 8 weeks [181]. Placental MSC-EVs caused dog bite wounds to heal within 10 days with minimal scarring, while plant-derived rose stem cell-EVs caused complete healing of vulvar wounds in patients with Behçet’s disease within 4 weeks [182, 183]. Specifically concerning ADSC-Exos, Kwon et al. reported that topical application of ADSC-Exos after laser treatment significantly improved acne scar remodeling [184]. In skin aging studies, intradermal injection of ADSC-Exos significantly improved skin firmness, wrinkles, and hydration without adverse effects [185]. Importantly, there were no treatment-related adverse events in all seven studies, whether the trauma was acute, chronic, or caused by surgical or other pathological factors. Local surface application, subcutaneous and intradermal injections, and multiple routes of administration were also safe and effective. These safety data and consistent efficacy in challenging cases demonstrate the feasibility of exosome-based therapies for clinical translation. Three clinical trials investigating exosomes/EVs in burns (NCT05078385), dystrophic epidermolysis bullosa (NCT04173650), and venous ulcers (NCT04652531) have been registered on ClinicalTrials.gov, and no results have been published. Collectively, these 7 clinical studies presented in Table 3 highlight the emerging prominence of exosomes/EVs in the field of wound healing and tissue regeneration. Although the results of clinical trials specifically evaluating ADSC-Exos for wound healing are not yet available, their well-documented biological properties in preclinical studies strongly suggest the great potential for future applications of ADSC-Exos in promoting wound healing (Table 3).

**Table 3:** Clinical trials of exosomes in wound healing and tissue regeneration

Row	ID (clinical trials)/Phase	The source of exosomes/EVs	Conditions	Control Group	Treatment Regimen	Outcomes	Author/Year/Reference
1	ACTRN12620000944932/Phase I	Human allogeneic platelet-derived EVs (pEVs)	Skin punch biopsy-induced wounds (healthy volunteers)	Placebo (formulation buffer)	11 adults (29 years); 100 µg pEVs in 340 µL; single subcutaneous injection adjacent to 4 mm punch biopsy wounds.	Safe, well-tolerated; no significant adverse events; no difference in healing time (22.8 ± 8.7 d) vs. placebo.	Johnson et al. 2023 [179]
2	Not applicable (Split-face RCT)	Human ADSC-Exos	Atrophic acne scars	Control gel (split-face)	25 patients (19–54 years); topical ADSC-Exos gel (9.78 × 10^9^ particles/mL post-laser, 1.63 × 10^9^ particles/mL subsequently); applied after fractional CO_2_ laser (3 sessions at 3-w intervals).	Significantly greater scar improvement (ECCA score reduction: 32.5% vs. 19.9%), reduced erythema, and shorter downtime vs. control.	Kwon et al. 2020 [184]
3	Not applicable (Clinical study)	ADSC-Exos	Skin aging	None (self-controlled)	72 females (34–68 years); intradermal injection a minimum of suspension until a visible wheal formation; single session.	Significant improvement in skin firmness, wrinkles, and hydration; no adverse effects.	Svolacchia et al. 2024 [185]
4	Case Report	Platelet-derived (PEP)	Nonhealing scalp wounds post-chemoradiation/surgery	None (self-controlled)	1 patient (60 years); topical application of collagen-PEP (0.2 mL frontal, 1 mL temporoparietal) + Fibrin-PEP (volume NS); 4 applications for each carrier over 7 mons.	Frontal wound: 100% healing; temporoparietal wound: 96% size reduction; no adverse effects.	Pumford et al. 2024 [180]
5	Case Series	Human placental MSCs	Post-procedural wound	None (self-controlled)	3 Patients (31–72 years); topical application of 5-12.5 × 10^9^ exosomes in 2.5-3 mL serum; single application post-procedure/trauma.	Laser patients, pain reduced significantly; erythema and swelling resolved rapidly. Dog bite, wound closed in 10 d; minimal scarring and well preserved sensory and motor function.	Peredo et al. 2024 [182]
6	Case Report	Rose stem cells	Surgical wounds in Behçet’s disease	None (self-controlled)	1 Patient (38 years); topical application of 2 layers of exosomes; intra-op + 1 w and 4 w post-op.	Complete healing at 4 w; the symmetrical size of labia majors, no wound complications, and restored sexual function at 6 w.	Elajami 2024 [183]
7	Case Report	Bone marrow-derived mesenchymal stem cells (ExoFlo^®^)	Recurrent right ischial pressure ulcer (5 × 4 × 5 cm)	None (self-controlled)	1 male (38 years); subcutaneous injection of 1 cc exosomes diluted in 4 cc saline; 6 injections over 8 w.	Complete ulcer healing achieved by 8 w; recurrence-free for 2 years until re-injury from prolonged pressure.	Messa et al. 2022 [181]

## Conclusions

**Fig. 4:**
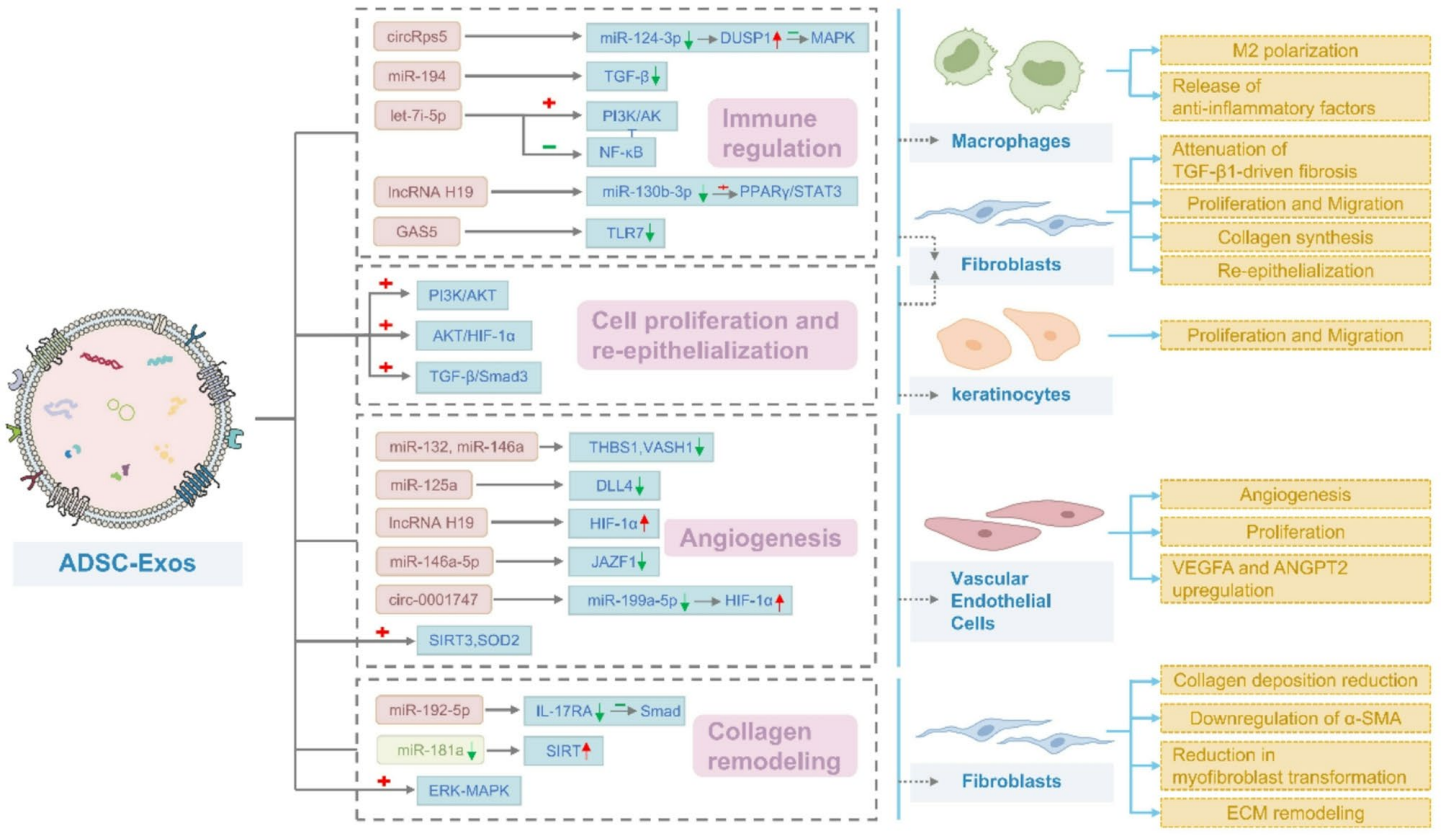
Roles and mechanisms of ADSC-Exos in wound healing. Adipose-derived stem cell exosomes (ADSC-Exos) facilitate wound healing by delivering a spectrum of bioactive molecules including metabolites, proteins, DNA, and various non-coding RNAs (ncRNAs). These exosomes enhance tissue repair through several key mechanisms: [1] Immune modulation: ADSC-Exos modulate inflammatory responses by altering macrophage polarization through ncRNAs like circRps5 and miR-146a, which orchestrate the shift from pro-inflammatory to reparative phenotypes [2]. Cell proliferation and migration: They promote fibroblast and keratinocyte activities essential for wound closure and epithelialization [3]. Angiogenesis: ADSC-Exos stimulate vascular endothelial cell proliferation and migration by delivering ncRNAs such as miR-132 and lncRNA H19, thereby enhancing neovascularization [4]. Collagen remodeling: ADSC-Exos regulate collagen synthesis and remodeling, crucial for tissue integrity and scar reduction, by modulating pathways such as the IL-17RA mediated by miR-192-5p. Collectively, these mechanisms contribute to the optimized healing process across different wound healing stages, from inflammation to tissue regeneration

Collectively, ADSC-Exos offer significant promise in regenerative medicine, particularly for enhancing wound healing. ADSC-Exos deliver a rich array of bioactive molecules, including ncRNAs and proteins, which modulate critical processes such as immune regulation, cell proliferation, angiogenesis, and collagen remodeling. Their ability to orchestrate these mechanisms makes them a potent tool for tissue repair. Recent advancements in engineering strategies, including genetic modification and preconditioning of ADSCs, have further enhanced the therapeutic potential of ADSC-Exos. Techniques such as hypoxic treatment and the incorporation of biomaterials like hydrogels have improved exosome stability and delivery efficiency, ensuring sustained release and targeted action at wound sites (Fig. 4). Despite these promising developments, challenges remain in the clinical translation of ADSC-Exos. Future research must focus on scalable production, standardized isolation methods, and comprehensive safety assessments. Additionally, exploring the biogenesis mechanisms and optimizing the cargo loading of exosomes will be crucial for refining their therapeutic efficacy. By utilizing interdisciplinary collaboration and adhering to rigorous ethical standards, ADSC-Exos can be effectively integrated into clinical practice, offering innovative solutions for patients with chronic and complex wounds.

## Data Availability

No datasets were generated or analysed during the current study.

## Abbreviations

ADSC-Exos: Adipose-derived stem cell exosomes
ADSCs: Adipose-derived stem cells
APP: Amyloid beta A4 protein
AMS: Amniotic membrane scaffold
Arg-1: Arginase-1
BM-MSCs: Bone marrow mesenchymal stem cells
CGRP: Calcitonin gene-related peptide
CCL2: C-C motif chemokine ligand 2
circRNAs: circular RNAs
e-circRps5: circRps5 overexpressed plasmid
CRP: C-reactive protein
DCs: Dendritic cells
DLL4: Delta-like 4
DDR2: Discoidin domain receptor 2
ESCRT: Endosomal sorting complex required for transport
EGF: Epidermal growth factor
ECM: Extracellular matrix
EVs: Extracellular vesicles
EXODUS: Exosome detection via the ultrafast-isolation system
FGF: Fibroblast growth factor
GMP: Good Manufacturing Practice
HSP: Heat shock protein
HDAC: Histone deacetylase
HSFs: Human scar-derived fibroblasts
hAAM: Human acellular amniotic membrane
HDFs: Human dermal fibroblasts
HUVECs: Human umbilical vein endothelial cells
Hpy-ADSC-Exos: Hypoxia-cultured ADSC-Exos
HIF-1α: Hypoxia-inducible factor-1α
ILVs: Intra-luminal vesicles
IFN-α: Interferon-α
IFN-γ: Interferon-γ
ITGB-1: Integrin beta-1
IL-10: Interleukin-10
FEP: Injectable thermosensitive adhesive multifunctional polysaccharide-based hydrogel
lncRNAs: Long non-coding RNAs
LPS: Lipopolysaccharide
M1: Macrophages 1
MHC: Major histocompatibility complex
MMPs: Matrix metalloproteinases
MSCs: Mesenchymal stem cells
miRNAs: microRNAs
MDFs: Mouse dermal fibroblasts
MVBs: Multi-vesicular bodies
ncRNAs: Non-coding RNAs
PTEN: Phosphatase and tensin homolog deleted on chromosome ten
PI3K: Phosphatidylinositol 3-kinase
PDGF: Platelet-derived growth factor
PCNA: Proliferating cell nuclear antigen
AKT: Protein kinase B
pEVs: Platelet-derived EVs
ROCK1: Rho-associated coiled-coil containing protein kinase 1
RBPs: RNA-binding proteins
ROS: Reactive oxygen species
SVF: Stromal vascular fraction
SIRT3: Sirtuin 3
SOD2: Superoxide dismutase 2
TLR7: Toll-like receptor 7
TNF-α: Tumor necrosis factor-α
TGF-β: Transforming growth factor-β
THBS1: Thrombospondin-1
VASH1: Vasohibin-1
VEGF: Vascular endothelial growth factor
XIST: X-inactive-specific transcript
α-SMA: α-smooth muscle actin

## References

[1] Ma H, Siu WS, Leung PC. The potential of MSC-Based Cell-Free therapy in wound Healing-A thorough literature review. Int J Mol Sci. 2023;24(11). 10.3390/ijms24119356 PMC10253384 37298306

[2] Luo H, Wang Z, Qi F, Wang D. Applications of human amniotic fluid stem cells in wound healing. Chin Med J (Engl). 2022;135(19):2272–81. 36535008 10.1097/CM9.0000000000002076 PMC9771343

[3] Menni A, Moysidis M, Tzikos G, Stavrou G, Tsetis JK, Shrewsbury AD et al. Looking for the ideal probiotic healing regime. Nutrients. 2023;15(13). 10.3390/nu15133055 PMC10346906 37447381

[4] Tripathi R, Knusel KD, Ezaldein HH, Honaker JS, Bordeaux JS, Scott JF. Incremental health care expenditure of chronic cutaneous ulcers in the united States. JAMA Dermatol. 2019;155(6):694–9. 30892572 10.1001/jamadermatol.2018.5942 PMC6563532

[5] Ma X, Wang A, Zhang X, Zhang J, Li J, Fu X, et al. Photo-crosslinking injectable photothermal antibacterial hydrogel based on quaternary ammonium grafted Chitosan and hyaluronic acid for infected wound healing. Mater Today Bio. 2024;29:101265. 40018434 10.1016/j.mtbio.2024.101265 PMC11866169

[6] Wang Z, Qin Y, Yang C, Wei X, Qian J, Tu S, et al. Conservative treatment of urinary fistula: case report. Exp Ther Med. 2022;24(2):491. 35837074 10.3892/etm.2022.11418 PMC9257753

[7] Feng Z, Wang S, Huang W, Bai W. A potential bilayer skin substitute based on electrospun silk-elastin-like protein nanofiber membrane covered with bacterial cellulose. Colloids Surf B Biointerfaces. 2024;234:113677. 38043505 10.1016/j.colsurfb.2023.113677

[8] Karakawa R, Yoshimatsu H, Fuse Y, Yano T. Multiple flap transfer for multiple local recurrence of soft tissue sarcoma. Med (Kaunas). 2023;59(8). 10.3390/medicina59081489 PMC10456343 37629779

[9] Yang Q, Fang D, Chen J, Hu S, Chen N, Jiang J, et al. LncRNAs associated with oxidative stress in diabetic wound healing: regulatory mechanisms and application prospects. Theranostics. 2023;13(11):3655–74. 37441585 10.7150/thno.85823 PMC10334824

[10] Freedman BR, Hwang C, Talbot S, Hibler B, Matoori S, Mooney DJ. Breakthrough treatments for accelerated wound healing. Sci Adv. 2023;9(20):eade7007. 37196080 10.1126/sciadv.ade7007 PMC10191440

[11] Qin Y, Ge G, Yang P, Wang L, Qiao Y, Pan G, et al. An update on Adipose-Derived stem cells for regenerative medicine: where challenge Meets opportunity. Adv Sci (Weinh). 2023;10(20):e2207334. 37162248 10.1002/advs.202207334 PMC10369252

[12] Yang S, Sun Y, Yan C. Recent advances in the use of extracellular vesicles from adipose-derived stem cells for regenerative medical therapeutics. J Nanobiotechnol. 2024;22(1):316. 10.1186/s12951-024-02603-4 PMC11157933 38844939

[13] Smakaj A, De Mauro D, Rovere G, Pietramala S, Maccauro G, Parolini O et al. Clinical application of adipose derived stem cells for the treatment of aseptic Non-Unions: current stage and future Perspectives-Systematic review. Int J Mol Sci. 2022;23(6). 10.3390/ijms23063057 PMC8950719 35328476

[14] Bydon M, Qu W, Moinuddin FM, Hunt CL, Garlanger KL, Reeves RK, et al. Intrathecal delivery of adipose-derived mesenchymal stem cells in traumatic spinal cord injury: phase I trial. Nat Commun. 2024;15(1):2201. 38561341 10.1038/s41467-024-46259-y PMC10984970

[15] Kouroupis D, Kaplan LD, Ricordi C, Best TM. Mesenchymal stem/stromal Cell-Derived small extracellular vesicles (MSC-sEVs): A promising treatment modality for diabetic foot ulcer. Bioeng (Basel). 2023;10(10). 10.3390/bioengineering10101140 PMC10604677 37892870

[16] Yan D, Song Y, Zhang B, Cao G, Zhou H, Li H, et al. Progress and application of adipose-derived stem cells in the treatment of diabetes and its complications. Stem Cell Res Ther. 2024;15(1):3. 38167106 10.1186/s13287-023-03620-0 PMC10763319

[17] Ren G, Peng Q, Fink T, Zachar V, Porsborg SR. Potency assays for human adipose-derived stem cells as a medicinal product toward wound healing. Stem Cell Res Ther. 2022;13(1):249. 35690872 10.1186/s13287-022-02928-7 PMC9188073

[18] Buzas EI. The roles of extracellular vesicles in the immune system. Nat Rev Immunol. 2023;23(4):236–50. 35927511 10.1038/s41577-022-00763-8 PMC9361922

[19] Pegtel DM, Gould SJ, Exosomes. Annu Rev Biochem. 2019;88:487–514. 31220978 10.1146/annurev-biochem-013118-111902

[20] Lai JJ, Chau ZL, Chen SY, Hill JJ, Korpany KV, Liang NW, et al. Exosome processing and characterization approaches for research and technology development. Adv Sci (Weinh). 2022;9(15):e2103222. 35332686 10.1002/advs.202103222 PMC9130923

[21] Amiri A, Bagherifar R, Ansari Dezfouli E, Kiaie SH, Jafari R, Ramezani R. Exosomes as bio-inspired nanocarriers for RNA delivery: Preparation and applications. J Transl Med. 2022;20(1):125. 35287692 10.1186/s12967-022-03325-7 PMC8919142

[22] Kalluri R, LeBleu VS. The biology, function, and biomedical applications of exosomes. Science. 2020;367(6478). 10.1126/science.aau6977 PMC7717626 32029601

[23] Al-Madhagi H. The landscape of exosomes biogenesis to clinical applications. Int J Nanomed. 2024;19:3657–75. 10.2147/IJN.S463296 PMC11048319 38681093

[24] Zeng N, Yan ZP, Chen XY, Ni GX. Infrapatellar fat pad and knee osteoarthritis. Aging Dis. 2020;11(5):1317–28. 33014539 10.14336/AD.2019.1116 PMC7505265

[25] Liu J, Zhang Y, Tian Y, Huang W, Tong N, Fu X. Integrative biology of extracellular vesicles in diabetes mellitus and diabetic complications. Theranostics. 2022;12(3):1342–72. 35154494 10.7150/thno.65778 PMC8771544

[26] Li P, Liu Z, Xie Y, Gu H, Dai Q, Yao J, et al. Serum exosomes attenuate H(2)O(2)-Induced apoptosis in rat H9C2 cardiomyocytes via ERK1/2. J Cardiovasc Transl Res. 2019;12(1):37–44. 29404859 10.1007/s12265-018-9791-3

[27] Yu H, Wu Y, Zhang B, Xiong M, Yi Y, Zhang Q, et al. Exosomes derived from E2F1(-/-) Adipose-Derived stem cells promote skin wound healing via miR-130b-5p/TGFBR3 Axis. Int J Nanomed. 2023;18:6275–92. 10.2147/IJN.S431725 PMC10629453 37941530

[28] Xiong M, Zhang Q, Hu W, Zhao C, Lv W, Yi Y, et al. Exosomes from Adipose-Derived stem cells: the emerging roles and applications in tissue regeneration of plastic and cosmetic surgery. Front Cell Dev Biol. 2020;8:574223. 33015067 10.3389/fcell.2020.574223 PMC7511773

[29] Airuddin SS, Halim AS, Wan Sulaiman WA, Kadir R, Nasir NAM. Adipose-Derived Stem Cell: Treat or Trick Biomedicines. 2021;9(11). 10.3390/biomedicines9111624 PMC8615427 34829853

[30] Babu S, Krishnan M, Panneerselvam A, Chinnaiyan M. A comprehensive review on therapeutic application of mesenchymal stem cells in neuroregeneration. Life Sci. 2023;327:121785. 37196856 10.1016/j.lfs.2023.121785

[31] Glovinski PV, Herly M, Mathiasen AB, Svalgaard JD, Borup R, Talman MM, et al. Overcoming the bottleneck of platelet lysate supply in large-scale clinical expansion of adipose-derived stem cells: A comparison of fresh versus three types of platelet lysates from outdated Buffy coat-derived platelet concentrates. Cytotherapy. 2017;19(2):222–34. 27887865 10.1016/j.jcyt.2016.10.014

[32] Zhou R, Zheng B. [Research advance of on the support effect of adipose Tissue-Derived stem cell on hematopoietic stem/Progenitor cell–Review]. Zhongguo Shi Yan Xue Ye Xue Za Zhi. 2021;29(1):301–5. 33554839 10.19746/j.cnki.issn.1009-2137.2021.01.051

[33] Mikłosz A, Chabowski A. Adipose-derived mesenchymal stem cells therapy as a new treatment option for diabetes mellitus. J Clin Endocrinol Metab. 2023;108(8):1889–97. 36916961 10.1210/clinem/dgad142 PMC10348459

[34] Zhou C, Zhang B, Yang Y, Jiang Q, Li T, Gong J, et al. Stem cell-derived exosomes: emerging therapeutic opportunities for wound healing. Stem Cell Res Ther. 2023;14(1):107. 37101197 10.1186/s13287-023-03345-0 PMC10134577

[35] Chen S, Sun F, Qian H, Xu W, Jiang J. Preconditioning and engineering strategies for improving the efficacy of mesenchymal stem Cell-Derived exosomes in Cell-Free therapy. Stem Cells Int. 2022;2022:1779346. 35607400 10.1155/2022/1779346 PMC9124131

[36] Kang Y, Xu C, Meng L, Dong X, Qi M, Jiang D. Exosome-functionalized magnesium-organic framework-based scaffolds with osteogenic, angiogenic and anti-inflammatory properties for accelerated bone regeneration. Bioact Mater. 2022;18:26–41. 35387167 10.1016/j.bioactmat.2022.02.012 PMC8961306

[37] Robbins PD, Morelli AE. Regulation of immune responses by extracellular vesicles. Nat Rev Immunol. 2014;14(3):195–208. 24566916 10.1038/nri3622 PMC4350779

[38] Raiborg C, Stenmark H. The ESCRT machinery in endosomal sorting of ubiquitylated membrane proteins. Nature. 2009;458(7237):445–52. 19325624 10.1038/nature07961

[39] Chen R, Kang Z, Li W, Xu T, Wang Y, Jiang Q, et al. Extracellular vesicle surface display of αPD-L1 and αCD3 antibodies via engineered late domain-based scaffold to activate T-cell anti-tumor immunity. J Extracell Vesicles. 2024;13(7):e12490. 39051742 10.1002/jev2.12490 PMC11270581

[40] Stuffers S, Sem Wegner C, Stenmark H, Brech A. Multivesicular endosome biogenesis in the absence of ESCRTs. Traffic. 2009;10(7):925–37. 19490536 10.1111/j.1600-0854.2009.00920.x

[41] de Gassart A, Géminard C, Hoekstra D, Vidal M. Exosome secretion: the Art of reutilizing nonrecycled proteins? Traffic. 2004;5(11):896–903. 15479454 10.1111/j.1600-0854.2004.00223.x

[42] Zeng Q, Liu CH, Ampuero J, Wu D, Jiang W, Zhou L, et al. Circular RNAs in non-alcoholic fatty liver disease: functions and clinical significance. RNA Biol. 2024;21(1):1–15. 38113132 10.1080/15476286.2023.2290769 PMC10761141

[43] Xiong S, Zhang J, Zhao Z, Liu J, Yao C, Huang J. NORAD accelerates skin wound healing through extracellular vesicle transfer from hypoxic adipose derived stem cells: miR-524-5p pathway and pumilio protein mechanism. Int J Biol Macromol. 2024;279(Pt 4):135621. 39276896 10.1016/j.ijbiomac.2024.135621

[44] Qian L, Li B, Pi L, Fang B, Meng X. Hypoxic adipose stem cell-derived exosomes carrying high-abundant USP22 facilitate cutaneous wound healing through stabilizing HIF-1α and upregulating LncRNA H19. Faseb J. 2024;38(10):e23653. 38738548 10.1096/fj.202301403RR

[45] Liu X, Wang B. Adipose stem cell-derived exosomes promote wound healing by regulating the let-7i-5p/GAS7 axis. J Cosmet Dermatol. 2024;23(6):2279–87. 38429909 10.1111/jocd.16267

[46] He C, Zheng S, Luo Y, Wang B. Exosome theranostics: biology and translational medicine. Theranostics. 2018;8(1):237–55. 29290805 10.7150/thno.21945 PMC5743472

[47] Zhang J, Li S, Li L, Li M, Guo C, Yao J, et al. Exosome and Exosomal microrna: trafficking, sorting, and function. Genomics Proteom Bioinf. 2015;13(1):17–24. 10.1016/j.gpb.2015.02.001 PMC4411500 25724326

[48] Parolini I, Federici C, Raggi C, Lugini L, Palleschi S, De Milito A, et al. Microenvironmental pH is a key factor for exosome traffic in tumor cells. J Biol Chem. 2009;284(49):34211–22. 19801663 10.1074/jbc.M109.041152 PMC2797191

[49] Savina A, Furlán M, Vidal M, Colombo MI. Exosome release is regulated by a calcium-dependent mechanism in K562 cells. J Biol Chem. 2003;278(22):20083–90. 12639953 10.1074/jbc.M301642200

[50] Välimäki E, Cypryk W, Virkanen J, Nurmi K, Turunen PM, Eklund KK, et al. Calpain activity is essential for ATP-Driven unconventional Vesicle-Mediated protein secretion and inflammasome activation in human macrophages. J Immunol. 2016;197(8):3315–25. 27638862 10.4049/jimmunol.1501840

[51] King HW, Michael MZ, Gleadle JM. Hypoxic enhancement of exosome release by breast cancer cells. BMC Cancer. 2012;12:421. 22998595 10.1186/1471-2407-12-421 PMC3488584

[52] Szul T, Bratcher PE, Fraser KB, Kong M, Tirouvanziam R, Ingersoll S, et al. Toll-Like receptor 4 engagement mediates Prolyl endopeptidase release from airway epithelia via exosomes. Am J Respir Cell Mol Biol. 2016;54(3):359–69. 26222144 10.1165/rcmb.2015-0108OC PMC5455678

[53] Yang GH, Lee YB, Kang D, Choi E, Nam Y, Lee KH, et al. Overcome the barriers of the skin: exosome therapy. Biomater Res. 2021;25(1):22. 34217362 10.1186/s40824-021-00224-8 PMC8254055

[54] Gao C, Chen Y, Wen X, Han R, Qin Y, Li S, et al. Plant-derived exosome-like nanoparticles in tissue repair and regeneration. J Mater Chem B. 2025;13(7):2254–71. 39817682 10.1039/d4tb02394c

[55] Wu W, Zhang B, Wang W, Bu Q, Li Y, Zhang P, et al. Plant-Derived Exosome-Like nanovesicles in chronic wound healing. Int J Nanomed. 2024;19:11293–303. 10.2147/IJN.S485441 PMC11549884 39524918

[56] An Y, Lin S, Tan X, Zhu S, Nie F, Zhen Y, et al. Exosomes from adipose-derived stem cells and application to skin wound healing. Cell Prolif. 2021;54(3):e12993. 33458899 10.1111/cpr.12993 PMC7941238

[57] Cai F, Chen W, Zhao R, Liu Y. The capacity of exosomes derived from adipose-derived stem cells to enhance wound healing in diabetes. Front Pharmacol. 2023;14:1063458. 37808198 10.3389/fphar.2023.1063458 PMC10551633

[58] Song Y, You Y, Xu X, Lu J, Huang X, Zhang J, et al. Adipose-Derived mesenchymal stem Cell-Derived exosomes biopotentiated extracellular matrix hydrogels accelerate diabetic wound healing and skin regeneration. Adv Sci (Weinh). 2023;10(30):e2304023. 37712174 10.1002/advs.202304023 PMC10602544

[59] Xu L, Liu D, Yun HL, Zhang W, Ren L, Li WW, et al. Effect of adipose-derived stem cells exosomes cross-linked chitosan-αβ-glycerophosphate thermosensitive hydrogel on deep burn wounds. World J Stem Cells. 2025;17(2):102091. 40061261 10.4252/wjsc.v17.i2.102091 PMC11885946

[60] Raziyeva K, Kim Y, Zharkinbekov Z, Kassymbek K, Jimi S, Saparov A. Immunology of acute and chronic wound healing. Biomolecules. 2021;11(5). 10.3390/biom11050700 PMC8150999 34066746

[61] Wilgus TA, Roy S, McDaniel JC. Neutrophils and wound repair: positive actions and negative reactions. Adv Wound Care (New Rochelle). 2013;2(7):379–88. 24527354 10.1089/wound.2012.0383 PMC3763227

[62] D’Amico R, Cordaro M, Fusco R, Peritore AF, Genovese T, Gugliandolo E et al. Consumption of cashew (Anacardium occidentale L.) nuts counteracts oxidative stress and tissue inflammation in mild hyperhomocysteinemia in rats. Nutrients. 2022;14(7). 10.3390/nu14071474 PMC9002620 35406088

[63] Holland SD, Ramer MS. Microglial activating transcription factor 3 upregulation: an indirect target to attenuate inflammation in the nervous system. Front Mol Neurosci. 2023;16:1150296. 37033378 10.3389/fnmol.2023.1150296 PMC10076742

[64] Yu H, Xiong J, Qiu J, He X, Sheng H, Dai Q, et al. Type III secretion protein, pcrv, impairs Pseudomonas aeruginosa biofilm formation by increasing M1 Macrophage-Mediated Anti-bacterial activities. Front Microbiol. 2020;11:1971. 32903626 10.3389/fmicb.2020.01971 PMC7438568

[65] Petejova N, Martinek A, Zadrazil J, Klementa V, Pribylova L, Bris R, et al. Expression and 7-day time course of Circulating MicroRNAs in septic patients treated with nephrotoxic antibiotic agents. BMC Nephrol. 2022;23(1):111. 35305556 10.1186/s12882-022-02726-6 PMC8933949

[66] Toda M, Mizuguchi S, Minamiyama Y, Yamamoto-Oka H, Aota T, Kubo S, et al. Pirfenidone suppresses polarization to M2 phenotype macrophages and the fibrogenic activity of rat lung fibroblasts. J Clin Biochem Nutr. 2018;63(1):58–65. 30087545 10.3164/jcbn.17-111 PMC6064814

[67] Wang EY, Zhao Y, Okhovatian S, Smith JB, Radisic M. Intersection of stem cell biology and engineering towards next generation in vitro models of human fibrosis. Front Bioeng Biotechnol. 2022;10:1005051. 36338120 10.3389/fbioe.2022.1005051 PMC9630603

[68] Ge Z, Chen Y, Ma L, Hu F, Xie L. Macrophage polarization and its impact on idiopathic pulmonary fibrosis. Front Immunol. 2024;15:1444964. 39131154 10.3389/fimmu.2024.1444964 PMC11310026

[69] Huo S, Liu S, Liu Q, Xie E, Miao L, Meng X, et al. Copper-Zinc-Doped bilayer bioactive glasses loaded hydrogel with Spatiotemporal Immunomodulation supports MRSA-Infected wound healing. Adv Sci (Weinh). 2024;11(5):e2302674. 38037309 10.1002/advs.202302674 PMC10837387

[70] Yin D, Shen G. Exosomes from adipose-derived stem cells regulate macrophage polarization and accelerate diabetic wound healing via the circ-Rps5/miR-124-3p axis. Immun Inflamm Dis. 2024;12(6):e1274. 38888351 10.1002/iid3.1274 PMC11184652

[71] Xu Z, Tian Y, Hao L. Exosomal miR–194 from adipose–derived stem cells impedes hypertrophic Scar formation through targeting TGF–β1. Mol Med Rep. 2024;30(6). 10.3892/mmr.2024.13340 PMC11465438 39329201

[72] Mahmoudi M, Taghavi-Farahabadi M, Rezaei N, Hashemi SM. Comparison of the effects of adipose tissue mesenchymal stromal cell-derived exosomes with conditioned media on neutrophil function and apoptosis. Int Immunopharmacol. 2019;74:105689. 31207404 10.1016/j.intimp.2019.105689

[73] Knoedler S, Knoedler L, Kauke-Navarro M, Rinkevich Y, Hundeshagen G, Harhaus L, et al. Regulatory T cells in skin regeneration and wound healing. Mil Med Res. 2023;10(1):49. 37867188 10.1186/s40779-023-00484-6 PMC10591349

[74] Blazquez R, Sanchez-Margallo FM, de la Rosa O, Dalemans W, Alvarez V, Tarazona R, et al. Immunomodulatory potential of human adipose mesenchymal stem cells derived exosomes on in vitro stimulated T cells. Front Immunol. 2014;5:556. 25414703 10.3389/fimmu.2014.00556 PMC4220146

[75] Kouroupis D, Kaplan LD, Best TM. Human infrapatellar fat pad mesenchymal stem cells show Immunomodulatory Exosomal signatures. Sci Rep. 2022;12(1):3609. 35246587 10.1038/s41598-022-07569-7 PMC8897449

[76] Liebmann K, Castillo MA, Jergova S, Best TM, Sagen J, Kouroupis D. Modification of mesenchymal stem/stromal Cell-Derived small extracellular vesicles by calcitonin gene related peptide (CGRP) antagonist: potential implications for inflammation and pain reversal. Cells. 2024;13(6). 10.3390/cells13060484 PMC10969778 38534328

[77] Zaki A, Ali MS, Hadda V, Ali SM, Chopra A, Fatma T. Long non-coding RNA (lncRNA): A potential therapeutic target in acute lung injury. Genes Dis. 2022;9(5):1258–68. 35873025 10.1016/j.gendis.2021.07.004 PMC9293716

[78] Xiao S, Xiao C, Miao Y, Wang J, Chen R, Fan Z, et al. Human acellular amniotic membrane incorporating exosomes from adipose-derived mesenchymal stem cells promotes diabetic wound healing. Stem Cell Res Ther. 2021;12(1):255. 33926555 10.1186/s13287-021-02333-6 PMC8082232

[79] Heo JS, Kim S, Yang CE, Choi Y, Song SY, Kim HO. Human adipose mesenchymal stem Cell-Derived exosomes: A key player in wound healing. Tissue Eng Regen Med. 2021;18(4):537–48. 33547566 10.1007/s13770-020-00316-x PMC8325736

[80] Li M, Li L, Zheng J, Li Z, Li S, Wang K, et al. Liquid biopsy at the frontier in renal cell carcinoma: recent analysis of techniques and clinical application. Mol Cancer. 2023;22(1):37. 36810071 10.1186/s12943-023-01745-7 PMC9942319

[81] Li B, Qian L, Pi L, Meng X. A therapeutic role of Exosomal LncRNA H19 from adipose mesenchymal stem cells in cutaneous wound healing by triggering macrophage M2 polarization. Cytokine. 2023;165:156175. 36948039 10.1016/j.cyto.2023.156175

[82] Heo JS, Kim S. Human adipose mesenchymal stem cells modulate inflammation and angiogenesis through exosomes. Sci Rep. 2022;12(1):2776. 35177768 10.1038/s41598-022-06824-1 PMC8854709

[83] Patel RS, Impreso S, Lui A, Vidyarthi G, Albear P, Patel NA. Long noncoding RNA GAS5 contained in exosomes derived from human adipose stem cells promotes repair and modulates inflammation in a chronic dermal wound healing model. Biology (Basel). 2022;11(3). 10.3390/biology11030426 PMC8945809 35336800

[84] Martin P. Wound healing–aiming for perfect skin regeneration. Science. 1997;276(5309):75–81. 9082989 10.1126/science.276.5309.75

[85] Hu L, Wang J, Zhou X, Xiong Z, Zhao J, Yu R, et al. Exosomes derived from human adipose mensenchymal stem cells accelerates cutaneous wound healing via optimizing the characteristics of fibroblasts. Sci Rep. 2016;6:32993. 27615560 10.1038/srep32993 PMC5018733

[86] Zhang W, Bai X, Zhao B, Li Y, Zhang Y, Li Z, et al. Cell-free therapy based on adipose tissue stem cell-derived exosomes promotes wound healing via the PI3K/Akt signaling pathway. Exp Cell Res. 2018;370(2):333–42. 29964051 10.1016/j.yexcr.2018.06.035

[87] Zhang Y, Han F, Gu L, Ji P, Yang X, Liu M, et al. Adipose mesenchymal stem cell exosomes promote wound healing through accelerated keratinocyte migration and proliferation by activating the AKT/HIF-1α axis. J Mol Histol. 2020;51(4):375–83. 32556903 10.1007/s10735-020-09887-4

[88] Loot MA, Kenter SB, Au FL, van Galen WJ, Middelkoop E, Bos JD, et al. Fibroblasts derived from chronic diabetic ulcers differ in their response to stimulation with EGF, IGF-I, bFGF and PDGF-AB compared to controls. Eur J Cell Biol. 2002;81(3):153–60. 11998867 10.1078/0171-9335-00228

[89] Hsu HH, Wang AYL, Loh CYY, Pai AA, Kao HK. Therapeutic potential of exosomes derived from diabetic adipose stem cells in cutaneous wound healing of db/db mice. Pharmaceutics. 2022;14(6). 10.3390/pharmaceutics14061206 PMC9227821 35745779

[90] Eilken HM, Adams RH. Dynamics of endothelial cell behavior in sprouting angiogenesis. Curr Opin Cell Biol. 2010;22(5):617–25. 20817428 10.1016/j.ceb.2010.08.010

[91] Santos TS, Santos I, Pereira-Filho RN, Gomes SVF, Lima-Verde IB, Marques MN, et al. Histological evidence of wound healing improvement in rats treated with oral administration of hydroalcoholic extract of Vitis labrusca. Curr Issues Mol Biol. 2021;43(1):335–52. 34208147 10.3390/cimb43010028 PMC8929082

[92] Dong L, Li X, Leng W, Guo Z, Cai T, Ji X, et al. Adipose stem cells in tissue regeneration and repair: from bench to bedside. Regen Ther. 2023;24:547–60. 37854632 10.1016/j.reth.2023.09.014 PMC10579872

[93] Chen DH, Huang JR, Su SL, Chen Q, Wu BY. Therapeutic potential of mesenchymal stem cells for cerebral small vessel disease. Regen Ther. 2024;25:377–86. 38414558 10.1016/j.reth.2023.11.002 PMC10899004

[94] Lyu K, Liu T, Chen Y, Lu J, Jiang L, Liu X, et al. A cell-free treatment for tendon injuries: adipose stem cell-derived exosomes. Eur J Med Res. 2022;27(1):75. 35643543 10.1186/s40001-022-00707-x PMC9148514

[95] Liang X, Zhang L, Wang S, Han Q, Zhao RC. Exosomes secreted by mesenchymal stem cells promote endothelial cell angiogenesis by transferring miR-125a. J Cell Sci. 2016;129(11):2182–9. 27252357 10.1242/jcs.170373

[96] Che D, Xiang X, Xie J, Chen Z, Bao Q, Cao D. Exosomes derived from adipose stem cells enhance angiogenesis in diabetic wound via miR-146a-5p/JAZF1 Axis. Stem Cell Rev Rep. 2024;20(4):1026–39. 38393667 10.1007/s12015-024-10685-8 PMC11087353

[97] Wang Z, Feng C, Liu H, Xia Y, Shan M, Hao Y. Hypoxia-induced adipose derived stem cells-derived exosomes promote diabetic wound healing through circ-0001747/miR-199a-5p/HIF-1α axis. Arch Dermatol Res. 2025;317(1):456. 39987303 10.1007/s00403-025-03921-9

[98] Deng L, Du C, Song P, Chen T, Rui S, Armstrong DG, et al. The role of oxidative stress and antioxidants in diabetic wound healing. Oxid Med Cell Longev. 2021;2021:8852759. 33628388 10.1155/2021/8852759 PMC7884160

[99] Zhang Y, Bai X, Shen K, Luo L, Zhao M, Xu C et al. Exosomes derived from adipose mesenchymal stem cells promote diabetic chronic wound healing through SIRT3/SOD2. Cells. 2022;11(16). 10.3390/cells11162568 PMC9406299 36010644

[100] Gurtner GC, Werner S, Barrandon Y, Longaker MT. Wound repair and regeneration. Nature. 2008;453(7193):314–21. 18480812 10.1038/nature07039

[101] Juin SK, Ouseph R, Gondim DD, Jala VR, Sen U. Diabetic nephropathy and gaseous modulators. Antioxid (Basel). 2023;12(5). 10.3390/antiox12051088 PMC10215699 37237955

[102] Finnerty CC, Jeschke MG, Branski LK, Barret JP, Dziewulski P, Herndon DN. Hypertrophic scarring: the greatest unmet challenge after burn injury. Lancet. 2016;388(10052):1427–36. 27707499 10.1016/S0140-6736(16)31406-4 PMC5380137

[103] Li Y, Zhang J, Shi J, Liu K, Wang X, Jia Y, et al. Exosomes derived from human adipose mesenchymal stem cells attenuate hypertrophic Scar fibrosis by miR-192-5p/IL-17RA/Smad axis. Stem Cell Res Ther. 2021;12(1):221. 33789737 10.1186/s13287-021-02290-0 PMC8010995

[104] Wei Y, Wang T, Zhang N, Ma Y, Shi S, Zhang R, et al. LncRNA TRHDE-AS1 inhibit the Scar fibroblasts proliferation via miR-181a-5p/PTEN axis. J Mol Histol. 2021;52(2):419–26. 33675502 10.1007/s10735-021-09968-y PMC8012339

[105] Chen J, Yu W, Xiao C, Su N, Han Y, Zhai L, et al. Exosome from adipose-derived mesenchymal stem cells attenuates Scar formation through microRNA-181a/SIRT1 axis. Arch Biochem Biophys. 2023;746:109733. 37652148 10.1016/j.abb.2023.109733

[106] Vuong TT, Rønning SB, Ahmed TAE, Brathagen K, Høst V, Hincke MT, et al. Processed eggshell membrane powder regulates cellular functions and increase MMP-activity important in early wound healing processes. PLoS ONE. 2018;13(8):e0201975. 30080894 10.1371/journal.pone.0201975 PMC6078314

[107] Wang L, Hu L, Zhou X, Xiong Z, Zhang C, Shehada HMA, et al. Exosomes secreted by human adipose mesenchymal stem cells promote scarless cutaneous repair by regulating extracellular matrix remodelling. Sci Rep. 2017;7(1):13321. 29042658 10.1038/s41598-017-12919-x PMC5645460

[108] Qin X, He J, Wang X, Wang J, Yang R, Chen X. The functions and clinical application potential of exosomes derived from mesenchymal stem cells on wound repair: a review of recent research advances. Front Immunol. 2023;14:1256687. 37691943 10.3389/fimmu.2023.1256687 PMC10486026

[109] Wang J, Yi Y, Zhu Y, Wang Z, Wu S, Zhang J, et al. [Effects of adipose-derived stem cell released exosomes on wound healing in diabetic mice]. Zhongguo Xiu Fu Chong Jian Wai Ke Za Zhi. 2020;34(1):124–31. 31939247 10.7507/1002-1892.201903058 PMC8171827

[110] An Y, Huang F, Tan X, Zhu S, Zhen Y, Shang Y, et al. Exosomes of adipose Tissue-Derived stem cells promote wound healing by sponging miR-17-5p and inducing autophagy protein Ulk1. Plast Reconstr Surg. 2023;151(5):1016–28. 36729201 10.1097/PRS.0000000000010083

[111] Pi L, Yang L, Fang BR, Meng XX, Qian L. LncRNA MALAT1 from human adipose-derived stem cell exosomes accelerates wound healing via miR-378a/FGF2 axis. Regen Med. 2022;17(9):627–41. 35822640 10.2217/rme-2021-0170

[112] Croissant C, Tuariihionoa A, Bacou M, Souleyreau W, Sala M, Henriet E, et al. DDR1 and DDR2 physical interaction leads to signaling interconnection but with possible distinct functions. Cell Adh Migr. 2018;12(4):324–34. 29616590 10.1080/19336918.2018.1460012 PMC6363034

[113] Cao W, Feng Y. LncRNA XIST promotes extracellular matrix synthesis, proliferation and migration by targeting miR-29b-3p/COL1A1 in human skin fibroblasts after thermal injury. Biol Res. 2019;52(1):52. 31540582 10.1186/s40659-019-0260-5 PMC6754631

[114] Zhu J, Quan H. Adipose-derived stem cells-derived exosomes facilitate cutaneous wound healing by delivering XIST and restoring discoidin domain receptor 2. Cytokine. 2022;158:155981. 35952595 10.1016/j.cyto.2022.155981

[115] Zhang M, Hu S, Liu L, Dang P, Liu Y, Sun Z, et al. Engineered exosomes from different sources for cancer-targeted therapy. Signal Transduct Target Ther. 2023;8(1):124. 36922504 10.1038/s41392-023-01382-y PMC10017761

[116] Lu S, Lu L, Liu Y, Li Z, Fang Y, Chen Z, et al. Native and engineered extracellular vesicles for wound healing. Front Bioeng Biotechnol. 2022;10:1053217. 36568307 10.3389/fbioe.2022.1053217 PMC9780283

[117] Xiang H, Ding P, Qian J, Lu E, Sun Y, Lee S, et al. Exosomes derived from minor salivary gland mesenchymal stem cells: a promising novel exosome exhibiting pro-angiogenic and wound healing effects similar to those of adipose-derived stem cell exosomes. Stem Cell Res Ther. 2024;15(1):462. 39627883 10.1186/s13287-024-04069-5 PMC11616330

[118] Yang X, Wang J, Guo SL, Fan KJ, Li J, Wang YL, et al. miR-21 promotes keratinocyte migration and re-epithelialization during wound healing. Int J Biol Sci. 2011;7(5):685–90. 21647251 10.7150/ijbs.7.685 PMC3107477

[119] Bai L, Liang R, Yang Y, Hou X, Wang Z, Zhu S, et al. MicroRNA-21 regulates PI3K/Akt/mTOR signaling by targeting TGFβI during skeletal muscle development in pigs. PLoS ONE. 2015;10(5):e0119396. 25950587 10.1371/journal.pone.0119396 PMC4423774

[120] Yang C, Luo L, Bai X, Shen K, Liu K, Wang J, et al. Highly-expressed micoRNA-21 in adipose derived stem cell exosomes can enhance the migration and proliferation of the HaCaT cells by increasing the MMP-9 expression through the PI3K/AKT pathway. Arch Biochem Biophys. 2020;681:108259. 31926164 10.1016/j.abb.2020.108259

[121] Rau CS, Yang JC, Chen YC, Wu CJ, Lu TH, Tzeng SL, et al. Lipopolysaccharide-induced microRNA-146a targets CARD10 and regulates angiogenesis in human umbilical vein endothelial cells. Toxicol Sci. 2014;140(2):315–26. 24863965 10.1093/toxsci/kfu097

[122] Yu H, Wang Y, Wang D, Yi Y, Liu Z, Wu M, et al. Landscape of the epigenetic regulation in wound healing. Front Physiol. 2022;13:949498. 36035490 10.3389/fphys.2022.949498 PMC9403478

[123] Si Q, Wu L, Pang D, Jiang P. Exosomes in brain diseases: pathogenesis and therapeutic targets. MedComm (2020). 2023;4(3):e287. 37313330 10.1002/mco2.287 PMC10258444

[124] Qiu J, Shu C, Li X, Ye C, Zhang WC. Exosomes from linc00511-overexpressing ADSCs accelerates angiogenesis in diabetic foot ulcers healing by suppressing PAQR3-induced Twist1 degradation. Diabetes Res Clin Pract. 2021;180:109032. 34461141 10.1016/j.diabres.2021.109032

[125] Liang ZH, Pan NF, Lin SS, Qiu ZY, Liang P, Wang J, et al. Exosomes from mmu_circ_0001052-modified adipose-derived stem cells promote angiogenesis of DFU via miR-106a-5p and FGF4/p38MAPK pathway. Stem Cell Res Ther. 2022;13(1):336. 35870977 10.1186/s13287-022-03015-7 PMC9308214

[126] Li X, Xie X, Lian W, Shi R, Han S, Zhang H, et al. Exosomes from adipose-derived stem cells overexpressing Nrf2 accelerate cutaneous wound healing by promoting vascularization in a diabetic foot ulcer rat model. Exp Mol Med. 2018;50(4):1–14. 29651102 10.1038/s12276-018-0058-5 PMC5938041

[127] Yang G, Waheed S, Wang C, Shekh M, Li Z, Wu J. Exosomes and their bioengineering strategies in the cutaneous wound healing and related complications: current knowledge and future perspectives. Int J Biol Sci. 2023;19(5):1430–54. 37056923 10.7150/ijbs.80430 PMC10086759

[128] Ogawa M, Udono M, Teruya K, Uehara N, Katakura Y. Exosomes derived from Fisetin-Treated keratinocytes mediate hair growth promotion. Nutrients. 2021;13(6). 10.3390/nu13062087 PMC8234638 34207142

[129] Hu C, Li L. Preconditioning influences mesenchymal stem cell properties in vitro and in vivo. J Cell Mol Med. 2018;22(3):1428–42. 29392844 10.1111/jcmm.13492 PMC5824372

[130] Han Y, Ren J, Bai Y, Pei X, Han Y. Exosomes from hypoxia-treated human adipose-derived mesenchymal stem cells enhance angiogenesis through VEGF/VEGF-R. Int J Biochem Cell Biol. 2019;109:59–68. 30710751 10.1016/j.biocel.2019.01.017

[131] Liang B, Liang JM, Ding JN, Xu J, Xu JG, Chai YM. Dimethyloxaloylglycine-stimulated human bone marrow mesenchymal stem cell-derived exosomes enhance bone regeneration through angiogenesis by targeting the akt/mtor pathway. Stem Cell Res Ther. 2019;10(1):335. 31747933 10.1186/s13287-019-1410-y PMC6869275

[132] Hu Y, Tao R, Chen L, Xiong Y, Xue H, Hu L, et al. Exosomes derived from pioglitazone-pretreated MSCs accelerate diabetic wound healing through enhancing angiogenesis. J Nanobiotechnol. 2021;19(1):150. 10.1186/s12951-021-00894-5 PMC8139165 34020670

[133] Liu W, Yu M, Xie D, Wang L, Ye C, Zhu Q, et al. Melatonin-stimulated MSC-derived exosomes improve diabetic wound healing through regulating macrophage M1 and M2 polarization by targeting the PTEN/AKT pathway. Stem Cell Res Ther. 2020;11(1):259. 32600435 10.1186/s13287-020-01756-x PMC7322868

[134] Yu M, Liu W, Li J, Lu J, Lu H, Jia W, et al. Exosomes derived from atorvastatin-pretreated MSC accelerate diabetic wound repair by enhancing angiogenesis via akt/enos pathway. Stem Cell Res Ther. 2020;11(1):350. 32787917 10.1186/s13287-020-01824-2 PMC7425015

[135] Shieh JS, Chin YT, Chiu HC, Hsieh YY, Cheng HR, Gu H et al. Bio-Pulsed stimulation effectively improves the production of avian mesenchymal stem Cell-Derived extracellular vesicles that enhance the bioactivity of skin fibroblasts and hair follicle cells. Int J Mol Sci. 2022;23(23). 10.3390/ijms232315010 PMC9740660 36499339

[136] Heo JS. Selenium-Stimulated exosomes enhance wound healing by modulating inflammation and angiogenesis. Int J Mol Sci. 2022;23(19). 10.3390/ijms231911543 PMC9570007 36232844

[137] Wu SC, Kuo PJ, Rau CS, Huang LH, Lin CW, Wu YC et al. Increased angiogenesis by exosomes secreted by Adipose-Derived stem cells upon lipopolysaccharide stimulation. Int J Mol Sci. 2021;22(16). 10.3390/ijms22168877 PMC8396299 34445582

[138] Estrada JC, Albo C, Benguría A, Dopazo A, López-Romero P, Carrera-Quintanar L, et al. Culture of human mesenchymal stem cells at low oxygen tension improves growth and genetic stability by activating Glycolysis. Cell Death Differ. 2012;19(5):743–55. 22139129 10.1038/cdd.2011.172 PMC3321628

[139] Wang J, Wu H, Peng Y, Zhao Y, Qin Y, Zhang Y, et al. Hypoxia adipose stem cell-derived exosomes promote high-quality healing of diabetic wound involves activation of PI3K/Akt pathways. J Nanobiotechnol. 2021;19(1):202. 10.1186/s12951-021-00942-0 PMC8261989 34233694

[140] Wu Z, Wang Z, Chen T, Wang D, Zhou F, Zhang G, et al. Dermal white adipose tissue: A new modulator in wound healing and regeneration. Regen Ther. 2025;28:115–25. 39717110 10.1016/j.reth.2024.11.015 PMC11665542

[141] Shi R, Jin Y, Zhao S, Yuan H, Shi J, Zhao H. Hypoxic ADSC-derived exosomes enhance wound healing in diabetic mice via delivery of circ-Snhg11 and induction of M2-like macrophage polarization. Biomed Pharmacother. 2022;153:113463. 36076572 10.1016/j.biopha.2022.113463

[142] Ju Y, Hu Y, Yang P, Xie X, Fang B. Extracellular vesicle-loaded hydrogels for tissue repair and regeneration. Mater Today Bio. 2023;18:100522. 36593913 10.1016/j.mtbio.2022.100522 PMC9803958

[143] Ma Y, Brocchini S, Williams GR. Extracellular vesicle-embedded materials. J Control Release. 2023;361:280–96. 37536545 10.1016/j.jconrel.2023.07.059

[144] Amengual-Tugores AM, Ráez-Meseguer C, Forteza-Genestra MA, Monjo M, Ramis JM. Extracellular Vesicle-Based hydrogels for wound healing applications. Int J Mol Sci. 2023;24(4). 10.3390/ijms24044104 PMC9967521 36835516

[145] Wang M, Chen J, Luo Y, Feng M, Yang Q, Tang Y, et al. Design strategies and application potential of multifunctional hydrogels for promoting angiogenesis. Int J Nanomed. 2024;19:12719–42. 10.2147/IJN.S495971 PMC11609418 39624117

[146] Safari B, Aghazadeh M, Davaran S, Roshangar L. Exosome-loaded hydrogels: A new cell-free therapeutic approach for skin regeneration. Eur J Pharm Biopharm. 2022;171:50–9. 34793943 10.1016/j.ejpb.2021.11.002

[147] Huang CC, Kang M, Shirazi S, Lu Y, Cooper LF, Gajendrareddy P, et al. 3D encapsulation and tethering of functionally engineered extracellular vesicles to hydrogels. Acta Biomater. 2021;126:199–210. 33741538 10.1016/j.actbio.2021.03.030 PMC8096714

[148] Hu H, Dong L, Bu Z, Shen Y, Luo J, Zhang H, et al. miR-23a-3p-abundant small extracellular vesicles released from gelma/nanoclay hydrogel for cartilage regeneration. J Extracell Vesicles. 2020;9(1):1778883. 32939233 10.1080/20013078.2020.1778883 PMC7480606

[149] Sang X, Zhao X, Yan L, Jin X, Wang X, Wang J, et al. Thermosensitive hydrogel loaded with primary Chondrocyte-Derived exosomes promotes cartilage repair by regulating macrophage polarization in osteoarthritis. Tissue Eng Regen Med. 2022;19(3):629–42. 35435577 10.1007/s13770-022-00437-5 PMC9130414

[150] Zhou Y, Zhang XL, Lu ST, Zhang NY, Zhang HJ, Zhang J, et al. Human adipose-derived mesenchymal stem cells-derived exosomes encapsulated in pluronic F127 hydrogel promote wound healing and regeneration. Stem Cell Res Ther. 2022;13(1):407. 35941707 10.1186/s13287-022-02980-3 PMC9358082

[151] Hu N, Cai Z, Jiang X, Wang C, Tang T, Xu T, et al. Hypoxia-pretreated ADSC-derived exosome-embedded hydrogels promote angiogenesis and accelerate diabetic wound healing. Acta Biomater. 2023;157:175–86. 36503078 10.1016/j.actbio.2022.11.057

[152] Liu Y, Liu Y, Zhao Y, Wu M, Mao S, Cong P, et al. Application of adipose mesenchymal stem cell-derived exosomes-loaded β-chitin nanofiber hydrogel for wound healing. Folia Histochem Cytobiol. 2022;60(2):167–78. 35645038 10.5603/FHC.a2022.0015

[153] Wu D, Tao S, Zhu L, Zhao C, Xu N. Chitosan hydrogel dressing loaded with adipose mesenchymal stem Cell-Derived exosomes promotes skin Full-Thickness wound repair. ACS Appl Bio Mater. 2024;7(2):1125–34. 38319146 10.1021/acsabm.3c01039

[154] Shafei S, Khanmohammadi M, Heidari R, Ghanbari H, Taghdiri Nooshabadi V, Farzamfar S, et al. Exosome loaded alginate hydrogel promotes tissue regeneration in full-thickness skin wounds: an in vivo study. J Biomed Mater Res A. 2020;108(3):545–56. 31702867 10.1002/jbm.a.36835

[155] Wang M, Wang C, Chen M, Xi Y, Cheng W, Mao C, et al. Efficient Angiogenesis-Based diabetic wound healing/skin reconstruction through bioactive antibacterial adhesive ultraviolet shielding nanodressing with exosome release. ACS Nano. 2019;13(9):10279–93. 31483606 10.1021/acsnano.9b03656

[156] Jung J, Kim JH, Re LK, Mell SL, Pugh CU, Jones et al. Effects of Androgen Deprivation Therapy on Prostate Cancer Outcomes According to Competing Event Risk: Secondary Analysis of a Phase 3 Randomised Trial. Eur Urol. In press. 10.1016/j.eururo.2023.01.020. Eur Urol. 2023;84(3):e73-e4. 10.1016/j.eururo.2023.04.037 37202316

[157] Shiekh PA, Singh A, Kumar A. Exosome laden oxygen releasing antioxidant and antibacterial cryogel wound dressing OxOBand alleviate diabetic and infectious wound healing. Biomaterials. 2020;249:120020. 32305816 10.1016/j.biomaterials.2020.120020

[158] Khalatbary AR, Omraninava M, Nasiry D, Akbari M, Taghiloo S, Poorhassan M, et al. Exosomes derived from human adipose mesenchymal stem cells loaded bioengineered three-dimensional amniotic membrane-scaffold-accelerated diabetic wound healing. Arch Dermatol Res. 2023;315(10):2853–70. 37644140 10.1007/s00403-023-02709-z

[159] Ge L, Wang K, Lin H, Tao E, Xia W, Wang F, et al. Engineered exosomes derived from miR-132-overexpresssing adipose stem cells promoted diabetic wound healing and skin reconstruction. Front Bioeng Biotechnol. 2023;11:1129538. 36937759 10.3389/fbioe.2023.1129538 PMC10014603

[160] Hoshino A, Costa-Silva B, Shen TL, Rodrigues G, Hashimoto A, Tesic Mark M, et al. Tumour exosome integrins determine organotropic metastasis. Nature. 2015;527(7578):329–35. 26524530 10.1038/nature15756 PMC4788391

[161] Hu JC, Zheng CX, Sui BD, Liu WJ, Jin Y. Mesenchymal stem cell-derived exosomes: A novel and potential remedy for cutaneous wound healing and regeneration. World J Stem Cells. 2022;14(5):318–29. 35722196 10.4252/wjsc.v14.i5.318 PMC9157601

[162] Nordberg RC, Loboa EG. Our fat future: translating adipose stem cell therapy. Stem Cells Transl Med. 2015;4(9):974–9. 26185256 10.5966/sctm.2015-0071 PMC4542878

[163] Wang J, Ma P, Kim DH, Liu BF, Demirci U. Towards Microfluidic-Based exosome isolation and detection for tumor therapy. Nano Today. 2021;37. 10.1016/j.nantod.2020.101066 PMC7990116 33777166

[164] Li P, Kaslan M, Lee SH, Yao J, Gao Z. Progress Exosome Isolation Techniques Theranostics. 2017;7(3):789–804. 28255367 10.7150/thno.18133 PMC5327650

[165] Carvalho AS, Baeta H, Henriques AFA, Ejtehadifar M, Tranfield EM, Sousa AL et al. Proteomic landscape of extracellular vesicles for diffuse large B-Cell lymphoma subtyping. Int J Mol Sci. 2021;22(20). 10.3390/ijms222011004 PMC8536203 34681663

[166] Vuerich R, Groppa E, Vodret S, Ring NAR, Stocco C, Bossi F, et al. Ischemic wound revascularization by the stromal vascular fraction relies on host-donor hybrid vessels. NPJ Regen Med. 2023;8(1):8. 36774354 10.1038/s41536-023-00283-6 PMC9922297

[167] Han W, Zhang H, Feng L, Dang R, Wang J, Cui C et al. The emerging role of exosomes in communication between the periphery and the central nervous system. MedComm (2020). 2023;4(6):e410. 10.1002/mco2.410 PMC10616655 37916034

[168] Zhang SH, Peng LL, Chen YF, Xu Y, Moradi V. Focusing on exosomes to overcome the existing bottlenecks of CAR-T cell therapy. Inflamm Regen. 2024;44(1):45. 39490997 10.1186/s41232-024-00358-x PMC11533312

[169] Sanchez-Manas JM, Perez de Gracia N, Perales S, Martinez-Galan J, Torres C, Real PJ. Potential clinical applications of extracellular vesicles in pancreatic cancer: exploring untapped opportunities from biomarkers to novel therapeutic approaches. Extracell Vesicles Circ Nucl Acids. 2024;5(2):180–200. 39698536 10.20517/evcna.2023.68 PMC11648502

[170] Kleinert M, Clemmensen C, Hofmann SM, Moore MC, Renner S, Woods SC, et al. Animal models of obesity and diabetes mellitus. Nat Rev Endocrinol. 2018;14(3):140–62. 29348476 10.1038/nrendo.2017.161

[171] Wu J, Chen LH, Sun SY, Li Y, Ran XW. Mesenchymal stem cell-derived exosomes: the dawn of diabetic wound healing. World J Diabetes. 2022;13(12):1066–95. 36578867 10.4239/wjd.v13.i12.1066 PMC9791572

[172] Ha DH, Kim SD, Lee J, Kwon HH, Park GH, Yang SH, et al. Toxicological evaluation of exosomes derived from human adipose tissue-derived mesenchymal stem/stromal cells. Regul Toxicol Pharmacol. 2020;115:104686. 32450131 10.1016/j.yrtph.2020.104686

[173] Zhao M, Shi J, Cai W, Liu K, Shen K, Li Z, et al. Advances on Graphene-Based nanomaterials and mesenchymal stem Cell-Derived exosomes applied in cutaneous wound healing. Int J Nanomed. 2021;16:2647–65. 10.2147/IJN.S300326 PMC8040697 33854313

[174] Lo YC, Lin CL, Fang WY, Lőrinczi B, Szatmári I, Chang WH et al. Effective activation by kynurenic acid and its aminoalkylated derivatives on M-Type K(+) current. Int J Mol Sci. 2021;22(3). 10.3390/ijms22031300 PMC7865226 33525680

[175] Gerber PA, Buhren BA, Schrumpf H, Homey B, Zlotnik A, Hevezi P. The top skin-associated genes: a comparative analysis of human and mouse skin transcriptomes. Biol Chem. 2014;395(6):577–91. 24497224 10.1515/hsz-2013-0279

[176] Dinh PC, Paudel D, Brochu H, Popowski KD, Gracieux MC, Cores J, et al. Inhalation of lung spheroid cell secretome and exosomes promotes lung repair in pulmonary fibrosis. Nat Commun. 2020;11(1):1064. 32111836 10.1038/s41467-020-14344-7 PMC7048814

[177] Wang Y, Cao Z, Wei Q, Ma K, Hu W, Huang Q, et al. VH298-loaded extracellular vesicles released from gelatin methacryloyl hydrogel facilitate diabetic wound healing by HIF-1α-mediated enhancement of angiogenesis. Acta Biomater. 2022;147:342–55. 35580827 10.1016/j.actbio.2022.05.018

[178] Kong Y, Wang Y, Yang Y, Hou Y, Yu J, Liu M, et al. Intra-articular injection of exosomes derived from different stem cells in animal models of osteoarthritis: a systematic review and meta- analysis. J Orthop Surg Res. 2024;19(1):834. 39696589 10.1186/s13018-024-05227-4 PMC11656911

[179] Johnson J, Law SQK, Shojaee M, Hall AS, Bhuiyan S, Lim MBL, et al. First-in-human clinical trial of allogeneic, platelet-derived extracellular vesicles as a potential therapeutic for delayed wound healing. J Extracell Vesicles. 2023;12(7):e12332. 37353884 10.1002/jev2.12332 PMC10290200

[180] Pumford AD, Staricha KL, Kunkel ET, Armstrong MF, Behfar A, Van Abel KM. Exosome Therapy for a Nonhealing Scalp Wound Following Chemoradiation and Surgical Therapy. Mayo Clin Proc. 2024;99(6):1006-12. 10.1016/j.mayocp.2024.04.011 38839179

[181] Messa GE, Tiongco RP, Lau FH. Treatment of a recurrent ischial ulcer with injected exosomes. J Surg Case Rep. 2022;2022(6):rjac271. 35774473 10.1093/jscr/rjac271 PMC9238301

[182] Peredo M, Shivananjappa S. Topical human mesenchymal stem Cell-Derived exosomes for acceleration of wound healing following tissue trauma and aesthetic procedures: A case series. J Drugs Dermatol. 2024;23(4):281–4. 38564379 10.36849/JDD.C7395

[183] Elajami MH. The usefulness of exosomes in accelerating healing and preventing complications in behçet’s disease: A case report. Cureus. 2024;16(11):e74476. 39600543 10.7759/cureus.74476 PMC11590039

[184] Kwon HH, Yang SH, Lee J, Park BC, Park KY, Jung JY, et al. Combination treatment with human adipose tissue stem Cell-derived exosomes and fractional CO2 laser for acne scars: A 12-week prospective, Double-blind, randomized, Split-face study. Acta Derm Venereol. 2020;100(18):adv00310. 33073298 10.2340/00015555-3666 PMC9309822

[185] Svolacchia F, Svolacchia L, Falabella P, Scieuzo C, Salvia R, Giglio F et al. Exosomes and Signaling Nanovesicles from the Nanofiltration of Preconditioned Adipose Tissue with Skin-B(^®^) in Tissue Regeneration and Antiaging: A Clinical Study and Case Report. Med (Kaunas). 2024;60(4). 10.3390/medicina60040670 PMC11051917 38674316

